# The role of finite strain kinematics in the natural fibre reinforcement of peat and other soft organic soils

**DOI:** 10.1038/s41598-025-25779-7

**Published:** 2025-11-25

**Authors:** Luis J. Parra-Gómez, Cristina Jommi, Stefano Muraro

**Affiliations:** 1 Department of Geoscience and Engineering, Delft University of Technology, Delft, 2628CN The Netherlands; 2 Department of Civil and Environmental Engineering, Politecnico di Milano, Milano, 20133 Italy

**Keywords:** Organic soils, Fibre reinforcement, Large strains, Soil fabric, Engineering, Materials science

## Abstract

Peat is a highly organic and fibrous soil that often presents significant challenges in geotechnical engineering due to its unconventional high compressibility, shearing resistance and anisotropy. While there is empirical evidence about the role of fibres, a mechanistic model that systematically explains their contribution to the response of the material is lacking. This study presents an experimental and numerical methodology to investigate the reinforcing role of fibres on the mechanical response of peat. An experimental campaign characterised the geometric and mechanical properties of individual peat fibres, highlighting size-dependent variability in tensile strength and stiffness that was modelled with a stochastic approach developed for fracture mechanics. Dynamic image analysis provided a detailed understanding of fibre size distributions, and a novel function was proposed to flexibly model fibre orientations in three dimensions. These findings informed the development of a numerical framework which incorporates large-strain kinematics to examine the effects of fibre reorientation and volumetric changes during material deformation. The results highlight the importance of fibre kinematics in shaping the stress-strain behaviour of peat and offer a framework for further exploration of the role of fibres in soft organic soils. The numerical results compared with laboratory data highlight that fibre reinforcement during shearing depends strongly on the previous strain history and the alignment between fibre orientation and the loading direction.

## Introduction

Histosol deposits like peat cover approximately 2.84% of the global land area and are distributed across all continents^1^. Managing them is pivotal in many endeavours as they play a substantial role in carbon capture, biodiversity, and water cycle regulation. At the same time, their intrinsic characteristics, particularly their high compressibility and ongoing decomposition, pose substantial challenges to infrastructure development. Structures built on highly organic soils are subject to significant settlements and deformations, and their total life-cycle costs escalate due to the requirement for frequent assessment and maintenance^2,3^. However, despite their engineering significance, there is, to date, no consensus on a comprehensive modelling framework of their mechanical behaviour.

Peat is a non-conventional material that is characterised by organic matter content (OMC) above 70%, gravimetric water content between 250% and 1000%, void ratio between 4.0 and 18.0 and fibre content between 10% and 80% by mass^4^. Peat is well known for its high compressibility and substantial rates of secondary compression^5–7^, which give it its well-known connotation of soft and often problematic soil. However, its soft nature is juxtaposed with its shear behaviour, which displays substantial hardening that allows the material to mobilise high friction angles sometimes exceeding the tension cut-off (TCO) surface^8–12^. This conjunction of properties implies that peat deposits routinely experience large displacements. Common testing procedures report natural volumetric strains larger than 100% during consolidation and deviatoric strains above 40% during shearing.

**Fig. 1:**
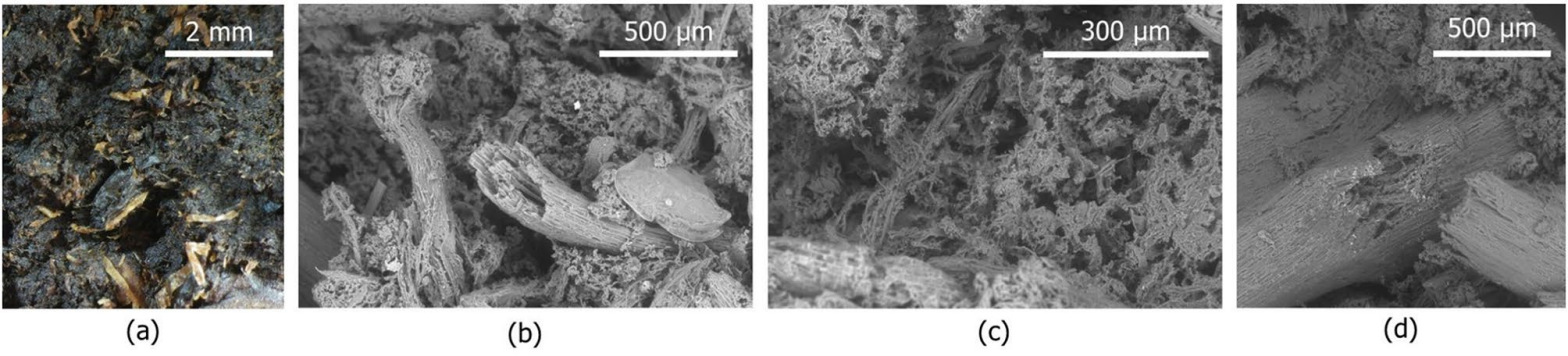
Microscopic observations of the fabric of a peat sample from the Netherlands. (**a**) Fibres and matrix as observed in the optical microscope; (**b**) Observations of the fibres and the matrix in the ESEM; (**c**) Inner structures of the peds as seen in the ESEM; (**d**) Fibre damage observed in the ESEM.

The peculiar properties of peat result from a complex and highly structured fabric composed of a multi-scale arrangement of fibrous plant organic matter with different sizes and decomposition levels. Figure 1 shows optical and environmental scanning electron microscope (ESEM) observations of the fabric of a natural peat sample from the Netherlands, and similar observations exist in the literature for other deposits ^13–15^. At the centimetric scale (Fig. 1a), the fabric appears as an arrangement of plant fibres embedded in an apparently homogeneous organic matrix. However, further zooming (Fig. 1b, and c) reveals that the organic peds are composed of smaller inter-crossing fibres that give rise to multiple levels of pores. Pores inside and between peds can be distinguished together with pores associated with the cellular structure of the fibres (Fig. 1b).

Volumetrically, the multi-scale fibre arrangement and porosity explain the high compressibility of the material, and the variable distribution of fibre orientations can intuitively explain its anisotropy. Upon shearing, the fibres, have been considered responsible for the strain hardening already observed in reconstituted samples ^12^ and the high shearing resistance attained at large strains. Researchers have proposed phenomenological approaches to account for the fibre reinforcement when defining the shear strength of the material^8,16–18^. However, these methods have been developed on empirical evidence and are often based on assumptions that can not be easily verified. Advanced constitutive models proposed in recent years ^12,19^ are able to capture several aspects of the material behaviour, but have been shown to have limitations in the presence of big fibres ^20^. Observations of the fabric have provided phenomenological explanations for the observed mechanical behaviour of peat, but have been by themselves insufficient for the derivation of a mechanical model.

Theories developed for fibre-reinforced or rooted soils^21–24^ have been used to attempt a quantitative link between fabric observations and sample response^20,25^. Peat fabric is idealised as a two-constituent system: a highly compressible organic matrix formed by the porous arrangement of small fibres and a reinforcing network of larger fibres that act as kinematic constraints. While promising, these theories face two important challenges. Firstly, contrary to man-made fibres, peat fibres are natural, highly heterogeneous materials that have variable and unknown sizes, orientations, and mechanical properties. Secondly, the large deformations of peat imply that fibres will change in volumetric proportion and orientation throughout loading. These models are often formulated for infinitesimal strains where these effects are not considered, and thus modifications are needed to make them applicable to soft soils like peat.

This work addresses both challenges through a combination of experimental and numerical techniques. Results from an experimental campaign characterising fibre tensile behaviour and size are presented and integrated into a single stochastic framework. Then, a mathematical construction to represent the distribution of fibre orientations in three-dimensional space is proposed and used with the experimental evidence to construct numerical analogues of the fibres within a soil sample. A large-strain numerical approach accounting for volume changes and fibre reorientation is proposed and the framework is used to quantify the role of fibre kinematics in the overall material response and compared with laboratory tests of natural peat samples.

## Experimental characterisation of peat fibres

To the best of the authors’ knowledge, no quantitative measurements of the mechanical properties of peat fibres have been reported to date. Existing studies provide some spatial measurements of fibre geometry and distribution derived from techniques such as microscope observations and X-ray tomography ^13,15,26^; however, these methods typically characterise a limited number of fibres and are constrained by the range of scales they can probe. To address this knowledge gap, an experimental campaign was undertaken to evaluate the geometric and mechanical properties of individual peat fibres isolated from the soil matrix.

### Tensile behaviour

**Fig. 2:**
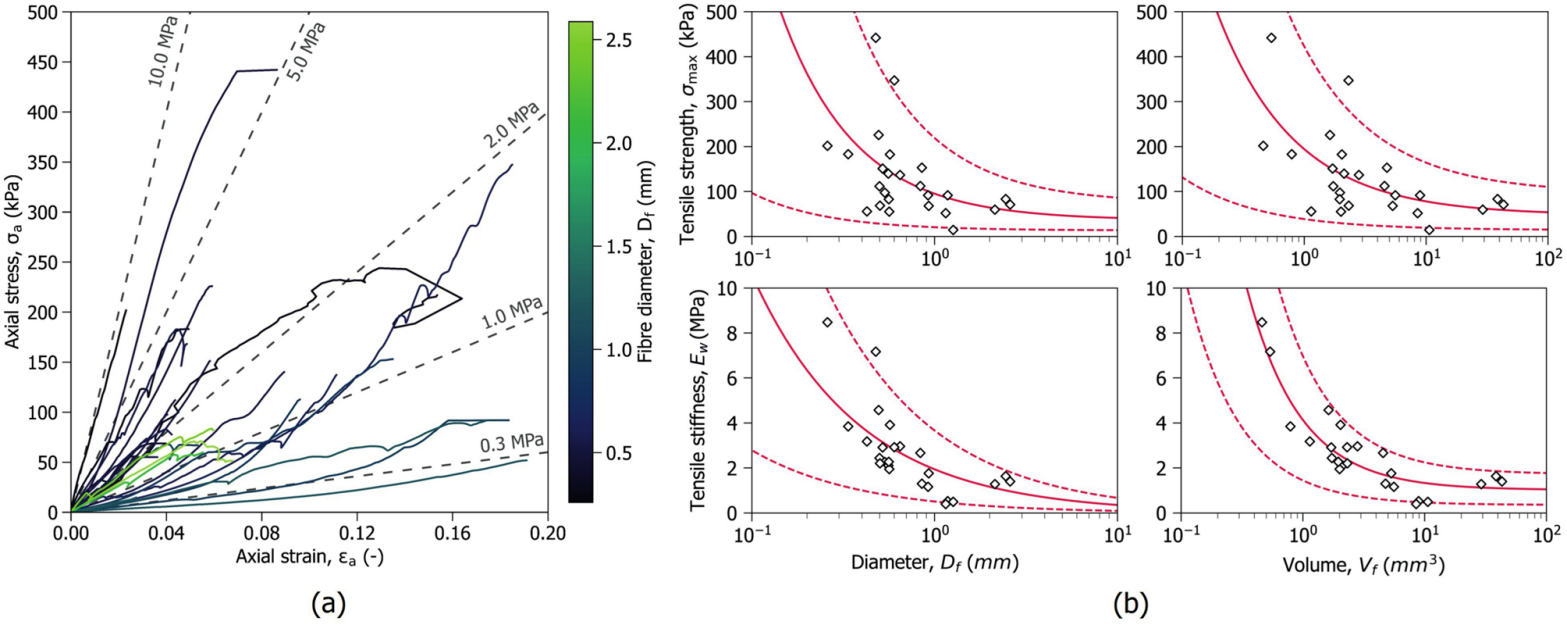
Results of single fibre tensile tests. (**a**) Stress-strain relationships; (**b**) Strength and stiffness with fibre size. Weibull fittings are shown in red lines, the solid line denotes the expected value, and the dashed ones show the 95% confidence band.

Twenty-five isolated peat fibres were mechanically tested in tension in saturated conditions, following the process described in the Methods section. Figure 2a shows the measured stress-strain relationships in terms of the logarithmic axial strain $\varepsilon _a$. Figure 2a reveals two distinct behaviours: smaller, straighter fibres exhibit a predominantly linear response, whereas larger, visually curlier fibres display a non-linear convex trend. The gradual increase in stiffness in curly fibres can be attributed to their resistance to straightening, which implies capacity to resist bending and, consequently, compression. As shown in Fig. 2b, fibre size is inversely correlated with both strength and stiffness, with the latter measured with reference to the constant value mobilised after the fibre is straightened. Table 1 summarises the testing results.

**Table 1:** Summary of fibre tension tests.

ID	L (mm)	Area (mm$^2$)	D (mm)	$E_t$(kPa)	$\sigma _{\text {max}}$ (kPa)	$\varepsilon _{max}$ (-)	Volume (mm$^3$*)*	Curliness (-)	CF (-)
03B	8.106	0.211	0.518	2919.4	151.1	0.058	1.708	C1	0.85
04A	8.831	0.195	0.499	2446.4	112.0	0.044	1.725	C0	1.00
04B	8.789	0.052	0.258	8479.3	201.8	0.023	0.460	C0	1.00
05A	8.780	0.223	0.532	2272.7	97.6	0.040	1.955	C0	1.00
05B	3.025	0.178	0.475	7179.2	442.1	0.086	0.537	C0	1.00
08A	8.000	0.142	0.426	3186.1	55.7	0.027	1.138	C1	0.62
08B	8.904	0.089	0.336	3855.9	183.1	0.049	0.789	C1	1.00
09B	8.530	0.190	0.492	4581.9	225.9	0.059	1.625	C1	0.86
10A	8.696	0.244	0.557	2108.6	140.1	0.089	2.121	C2	0.70
10B	8.322	0.547	0.835	2667.3	112.6	0.096	4.553	C2	0.36
11A	8.030	0.249	0.563	1953.0	55.2	0.029	1.999	C0	1.00
11B	7.918	0.246	0.560	2275.6	82.8	0.039	1.948	C0	1.00
14A	7.870	0.675	0.927	1766.6	68.3	0.040	5.312	C1	1.00
14B	8.550	0.328	0.646	2969.6	137.2	0.111	2.802	C2	0.36
15A	8.000	0.254	0.569	3924.3	182.8	0.045	2.032	C0	1.00
16A	8.141	0.283	0.600	2925.4	347.3	0.185	2.303	C2	0.62
20A	8.412	0.662	0.918	1167.5	91.9	0.183	5.567	C2	0.42
20B	8.370	0.567	0.850	1308.1	153.0	0.135	4.749	C1	0.84
20B*	11.711	0.198	0.502	2211.9	68.9	0.035	2.315	C0	1.00
22A	8.200	1.086	1.176	533.8	91.6	0.174	8.902	C0	1.00
22B	8.502	1.248	1.261	502.3	14.7	0.027	10.615	C2	1.00
30A	8.200	5.256	2.587	1399.0	71.0	0.054	43.098	C0	1.00
30B	8.140	3.569	2.132	1286.0	59.7	0.055	29.048	C0	1.00
31A	8.212	4.682	2.442	1644.2	83.5	0.059	38.453	C0	1.00
31B	8.200	1.035	1.148	402.5	51.9	0.191	8.487	C2	0.62

All tests concluded with a sudden brittle fracture, which was attributed to failure being governed by pre-existing weaknesses rather than the progressive deformation of the material. An example of one such weakness could be seen in Fig. 1d. The microscope observations of the post-test specimens revealed that many of the them had an inner fibre, with diameters between 0.3 mm and 0.5 mm, surrounded by a flat outer cover. In several cases, failure was triggered by breakage of the outer cover with the inner fibre (Fig. 11b) slipping before breaking (Fig. 11c). This mechanism provides a means for the fibres to sustain ductility after reaching their maximum strength.

The brittle failure and the diameter-dependent resistance of fibres are characteristics shared by many natural^27–29^ and man-made fibres^30,31^, and can be interpreted within the framework of fracture mechanics as described in the Methods section. The strength of peat fibres is stochastic and governed by flaws or weaknesses at discrete points that arise from various factors, including natural reductions in cross-sectional area, localised damage, and segments with higher decomposition levels. The probability of the fibre having a critical flaw increases with its size, explaining the inverse relationship between strength and diameter observed in Fig. 2b.

The experimental evidence indicates that the size-dependent variability also extends to the fibre stiffness. This behaviour can be linked to the observed fibre substructure and to the various causes of flaws it may have, such as variations in cross-sectional area or differences in decomposition rate depending on the fibre size. If the fibre components (e.g., elements of the outer layer and the inner core) are regarded as elastic elements acting in parallel, the stiffness trend could be attributed to the probability of damage occurring partially within them. Such damage would not necessarily lead to complete failure but would increase the apparent compliance of the fibre. For this reason, a stochastic framework was also adopted to describe the tensile stiffness. The tensile strength $\sigma _{\text {max}}$, and stiffness $E_w$, were modelled as three-point Weibull distributions, using either fibre volume or fibre diameter as size measure. Table 2 summarises the parameters obtained by maximum likelihood regression and the resulting fittings can be seen in Fig. 2b.

The average strength and stiffness predicted by the Weibull distribution follow an inverse power law, where the size of the fibre is raised to the power of the $\gamma _s$ parameter. By analogy, $\gamma _s$ can be taken to represent a fractal dimension of the size scaling; its magnitude provides insight into the mechanisms driving the decaying trend of the mechanical parameters. Values of $\gamma _s$ closer to 1.0 as reported in Table  2 suggest that stiffness decay is governed by the overall volume of the fibre, whereas strength decay is more closely linked to its diameter. This distinction suggests that phenomena affecting the overall mass of the fibre, such as the degree of organic decomposition, drive stiffness reduction while strength reduction may be linked to area reductions or localised damage.

**Table 2:** Weibull distribution parameters fitted to the tensile test data.

Var	Size	m (-)	$\sigma _0$ (kPa)	$\sigma _t$ (kPa)	$\sigma _m$ (kPa)	$V_0$ (*)	$\gamma _s$ (-)	Likelihood (-)
$\sigma _{\text {max}}$	Volume ( *mm*$^3$)	1.919	165.5	7.6	44.9	1.0	0.683	136.4
	Diameter ( *mm*)	1.659	66.0	10.1	29.0	1.0	1.063	138.9
$E_w$	Volume ( *mm*$^3$)	3.168	3477.3	0.0	1141.1	1.0	0.999	202.3
	Diameter ( *mm*)	2.529	2185.1	0.0	0.0	1.0	0.737	209.8

(*) $V_0$ has the same units as the size.

### Size distribution

Tensile characterisation indicates a strong correlation between mechanical properties and fibre size. However, obtaining a statistically representative characterisation of fibre sizes across a wide range of scales is challenging and, to the best of the authors’ knowledge, has not been previously reported in the literature. To address this gap, digital image analysis (DIA) was performed on isolated peat fibres dispersed in water, using samples from three distinct deposits in the Netherlands. Table 3 summarises the locations, sample types, and number of fibres analysed from each site. The measuring procedure is described in the Methods section.

**Table 3:** Summary of peat deposits investigated with DIA.

ID	Site	Samples	Fibres
LdB	Leendert de Boerspolder	5	1 227 871
Vst	Vlist	2	590 400
Zvd	Zegveld	2	576 745

**Fig. 3:**
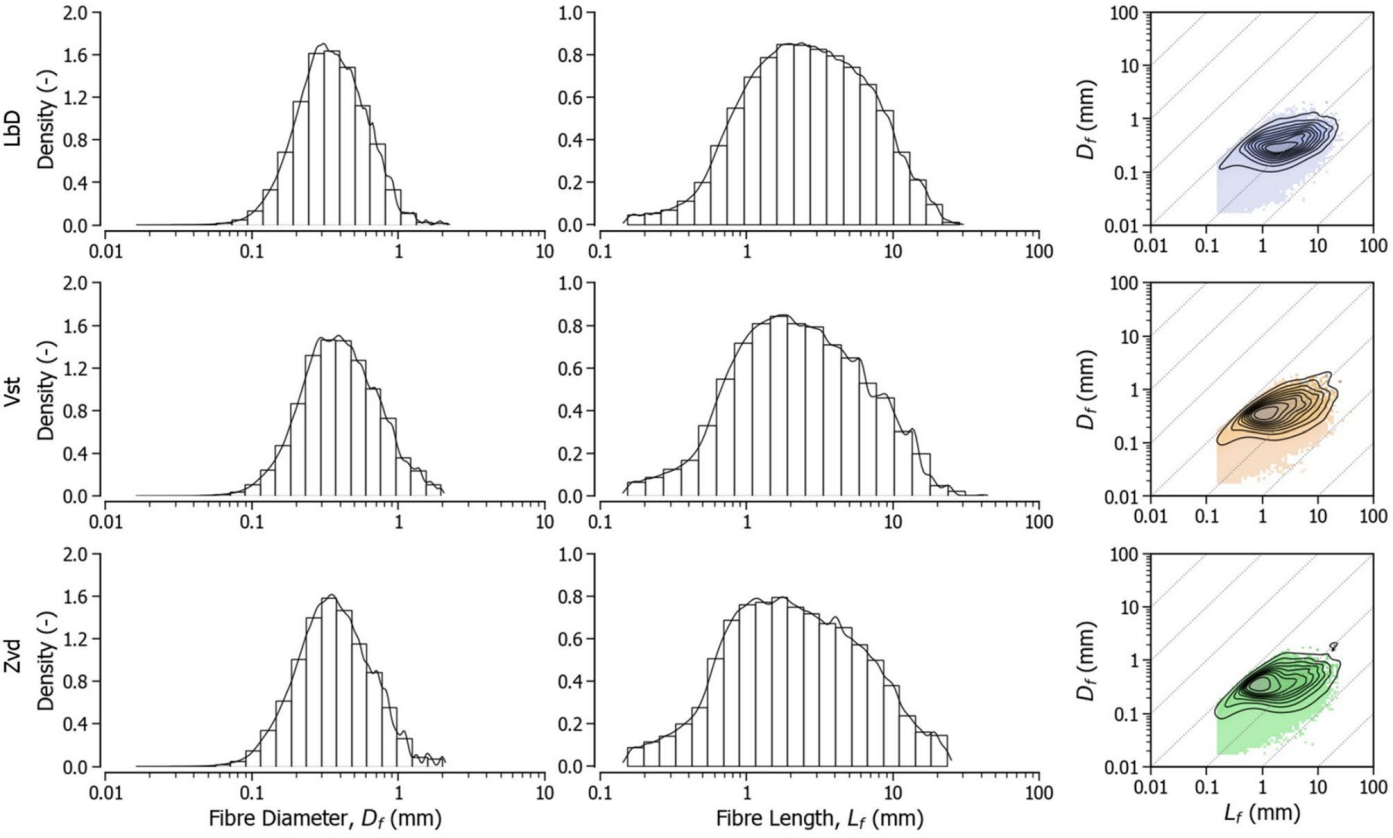
Fibre shape distributions obtained with by DIA. From left to right: Diameter, length and joint volume density distributions with density contours.

Figure 3 summarises the image analysis results for the aggregated samples at each deposit. Size characterisation is presented as volume density distributions, where the area under each histogram corresponds to the total observed fibre volume. Across all sites, the diameter distributions exhibit a log-normal component with mean values around 0.4 mm and standard deviations of approximately 0.25 mm, accounting for the majority of the measured fibre volume. Notably, the mean diameter of this component aligns with the inner fibre diameters observed under the microscope for fibres tested in tension. In the Vst and Zvd samples, small additional peaks are present between 1 mm and 2 mm in diameter, and at lengths exceeding 10 mm. These additional components contribute a smaller proportion to the total measured fibre volume (approximately 5 %).

The results provide valuable bounds to the size ranges that should be considered for mechanistic descriptions of peat fibre effects at the centimetric scale. However, it must be remarked that these bounds are subject to the biases introduced by the sample preparation process (i.e., fibre isolation) and the limitations of the measurement equipment which lead to the underestimation of the fibre length. These effects are discussed in the Methods section.

## A finite strain framework for the fibres

**Fig. 4:**
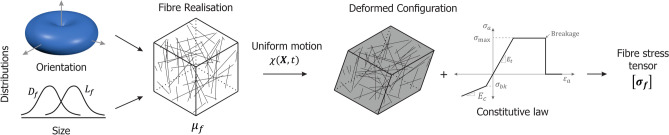
Conceptual framework for the finite strain approach.

Numerical simulations were performed using the parameter bounds obtained from the experimental results to evaluate the influence of fibre kinematics on the mechanical response of peat samples. The conceptual framework used for this analysis is illustrated in Fig. 4.

**Fig. 5:**
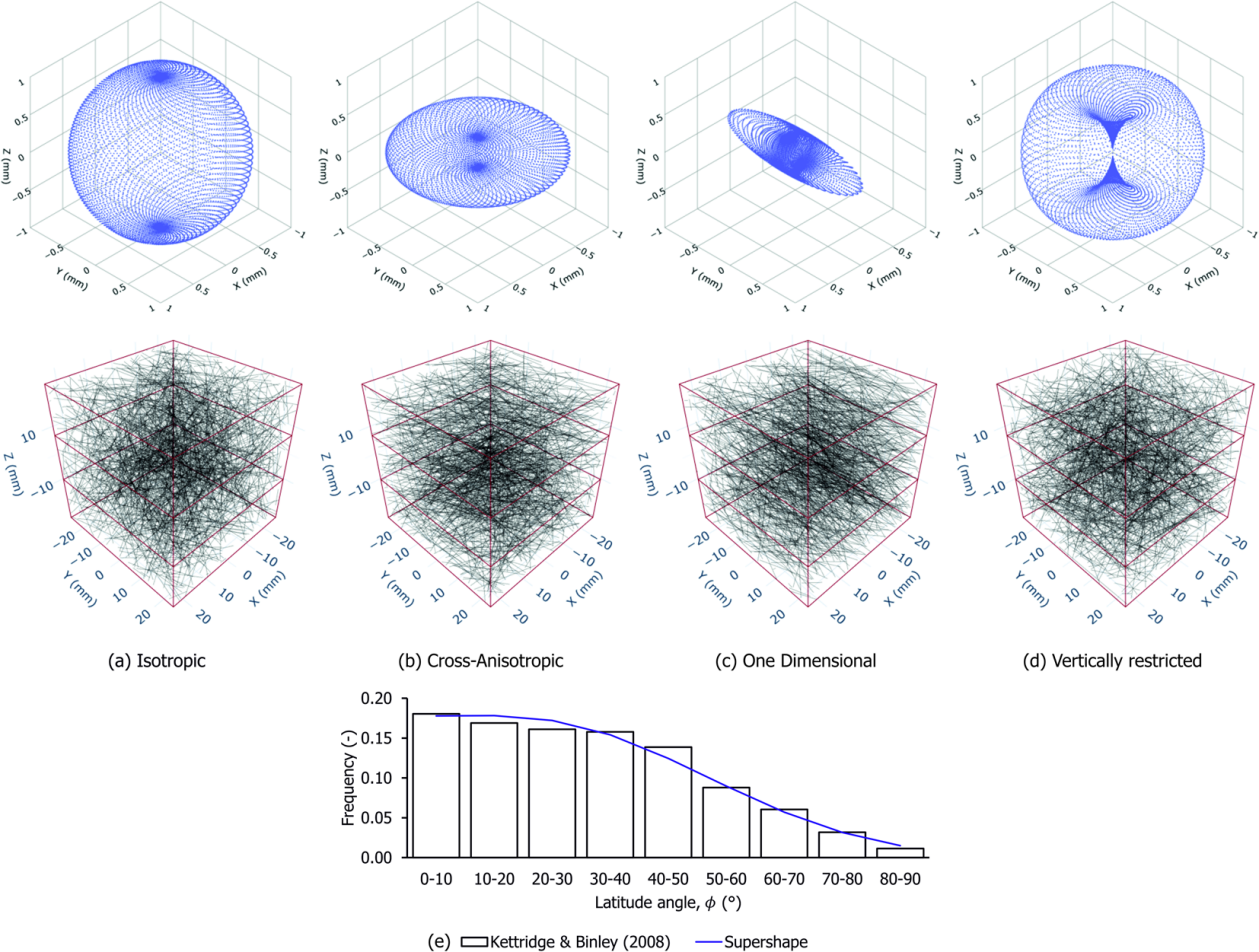
(**a**–**d**) Examples of fibre orientation distributions modelled with supershapes and corresponding fibre networks; (**e**) Vertically restricted distribution compared to data reported by Kettridge & Binley (2008)^26^.

Peat fibres were modelled explicitly as discrete, cylindrical one-dimensional elements with variable sizes, capable of sustaining axial tension and limited compression. Size and orientation distributions were used to generate a population of fibres representative of a volumetric fraction $\mu _f$ within a cubic Cartesian domain of volume $V$. Fibres evenly cover the domain comprising a representative elementary volume (REV) hereby referred as the sample. A threshold of 100 $\upmu \hbox {m}$ was chosen to distinguish between the organic matrix and the fibre network. The reasoning behind this distinction and the algorithm used to generate a random set of $n$ fibres with the desired characteristics and volumetric proportion is described in the Methods section. Figure 5 shows examples of the generated fibre networks.

The fibre arrangement is subjected to a continuous motion $\chi (\textbf{X}, t)$, which maps the material coordinate $\textbf{X}$ of a solid point to its position $\textbf{x}$ in the deformed configuration at time $t$ (Fig. 4). Although general finite strain motions may be spatially non-uniform and require solving boundary value problems, the present analysis considers only homogeneous deformation. This simplification allows the response of the sample to be interpreted as that of a representative material point (i.e., a Gauss point), subjected to a uniform strain increment. More complex loading paths can be recovered by integrating the constitutive response over successive increments, while spatial variability can be addressed through full-field numerical simulations, which lie outside the scope of this work.

The imposed motion allows for the computation of the increments of logarithmic strain for each fibre. These strain increments are then used to evaluate the corresponding axial stress through integration of the constitutive relation of the fibres. The resulting individual stresses are subsequently homogenised into a second-order stress tensor, $\varvec{\sigma _f}$, which provides a macroscopic representation of the overall mechanical effect of the fibres. This formulation naturally captures the reorientation of the fibre network and overall changes in volume and shape experienced by the sample . The role of kinematics is explored by comparing the stress tensors obtained from this formulation with those derived under infinitesimal strain assumptions, where all quantities are referred to the material configuration. Additional details on the finite strain implementation and stress homogenisation are provided in the Methods section.

Three characteristics must be specified for each fibre: its size (i.e., length and diameter), orientation, and mechanical properties. Each of these is detailed in the following subsections.

### Size distributions

Fibre sizes were modelled statistically based on the data obtained from the DIA experiments. The experimental results indicate that, in terms of number, the fibre diameter distribution is well-approximated by a log-normal distribution with a mean of 0.15 mm and a standard deviation of 0.1 mm. Based on the values observed in tensile tests, a deterministic aspect ratio of 50 was selected to define fibre lengths as a function of diameter. Diameters were sampled randomly from the log-normal distribution, and lengths were assigned accordingly.

### Orientation distributions

Fibre orientation distributions are commonly described by a surface defined over spherical coordinates, representing the orientation distribution density $\rho (\theta ,\phi )$. This function characterises the fibre volume density aligned with a given direction specified by the longitude $\theta$ and latitude $\phi$. Although $\rho (\theta ,\phi )$ can take any form that defines a closed surface symmetric with respect to the origin, it is convenient to scale it such that the enclosed volume equals the volumetric fibre content $\mu _f$, that is:

1 $$\begin{aligned} \int _{-\pi }^{\pi } \int _{-\pi /2}^{\pi /2} \rho (\theta ,\phi ) \cos {\phi } \ \textrm{d}\phi \textrm{d}\theta = \mu _f\ \ \ \ \text {and}\ \ \ \ \rho (\theta ,\phi ) = \rho (\theta + \pi , \phi + \pi ) \end{aligned}$$

Examples of such functions include those proposed by Michalowski and Čermák^32–34^, which simplify $\rho$ by neglecting variation with respect to longitude (i.e., axisymmetric assumption) and by enforcing small-strain assumptions. However, these simplifications are not appropriate for the analysis of large deformations, where fibre rearrangement cannot be neglected and initially cross-anisotropic orientations may evolve into general, fully three-dimensional distributions depending on the loading direction. A three-dimensional function suitable for numerical integration is therefore required to model peat fibres. One such formulation is the spherical product of two of the “supershape” functions proposed by Gielis^35^. This construction allows $\rho$ to be linked to the volume enclosed by a surface $\mathcal {S}$, obtained through a non-linear mapping of the unit direction vectors:

2 $$\begin{aligned} \rho (\theta ,\phi ) = \frac{1}{\Omega }\Vert \mathcal {S}(\theta ,\phi ) \Vert ^3 =\frac{1}{\Omega } \left\Vert \ \textbf{R}_{\alpha \beta }^{\theta \phi } \cdot r(\gamma ) r(\alpha ) \frac{\cos {\gamma }}{\cos {\beta }} \begin{bmatrix} \cos {\alpha } \cos {\beta } \\ \sin {\alpha } \cos {\beta } \\ \sin {\beta } \end{bmatrix}\right\Vert ^3 \end{aligned}$$

Here, $\textbf{R}^{\theta \phi }_{\alpha \beta }$ denotes an orthogonal rotation tensor that maps the Cartesian representation of direction vectors defined by the supershape angles $(\alpha , \beta )$ to those defined by the sample frame angles $(\theta , \phi )$. This rotation enables the surface to be freely oriented in three-dimensional space. The scaling factor $\Omega$ ensures that the enclosed volume satisfies Eq.(1). The angle $\gamma$ is defined through the relation $\tan {\gamma } = r(\alpha )\tan {\beta }$, and the functions $r(\alpha )$ and $r(\gamma )$ represent the radii of two two-dimensional supershapes defined over the angular coordinates $\alpha$ and $\gamma$, respectively. Each function is parametrised by a set of six shape parameters, denoted $\varvec{\Xi }_{\alpha }$ and $\varvec{\Xi }_{\gamma }$, and follows the general form:

3 $$\begin{aligned} r( \psi \mid \varvec{\Xi }_{\psi }) = \left[ \left| \frac{1}{a} \cos \left( \frac{m \psi }{4}\right) \right| ^{n_2} + \left| \frac{1}{b} \sin \left( \frac{m \psi }{4}\right) \right| ^{n_3} \right] ^{-1/n_1}, \quad \text {with} \quad \varvec{\Xi }_{\psi } = \left[ m, n_1, n_2, n_3, a, b\right] \end{aligned}$$

To ensure symmetry with respect to the origin, the parameter $m$ must be restricted to even natural numbers. The resulting distribution can represent a wide range of convex and concave shapes, while remaining straightforward to integrate numerically. In particular, concave shapes allow certain directions to be excluded, restricting fibre orientations in specific regions of the unit sphere. Figure 5 illustrates the versatility of the proposed function, showing examples of isotropic, elliptically cross-anisotropic, one-dimensional, and vertically restricted distributions, along with the corresponding fibre networks. The latter (Fig. 5d) was fitted to replicate the axisymmetric orientation density derived experimentally by Kettridge and Binley^26^ using X-ray tomography (see Fig. 5e). The resulting concave distribution is characterised by fibres predominantly aligned in the horizontal direction and restricted vertically, a pattern frequently observed in fibrous peat^13,15^. Table 4 lists the associated supershape parameters.

**Table 4:** Examples of supershape parameters used to describe different fibre orientation distributions.

**Distribution**	$\Xi _{\alpha }$						$\Xi _{\gamma }$					
	$m_{\alpha }$	$n_{1\alpha }$	$n_{2\alpha }$	$n_{3\alpha }$	$a_{\alpha }$	$b_{\alpha }$	$m_{\gamma }$	$n_{1\gamma }$	$n_{2\gamma }$	$n_{3\gamma }$	$a_{\gamma }$	$b_{\gamma }$
Isotropic	4	2	2	2	1	1	4	2	2	2	1	1
Cross-anisotropic	4	2	2	2	5	5	4	2	2	2	1	1
One dimensional	4	2	2	2	1	4	4	2	2	2	1	1
Vertically restricted	4	2	2	2	0.7	0.7	2	-0.8	8.5	1	0.1	0.1

### Constitutive response

Fibres were modelled as elastic-perfectly plastic, one-dimensional elements characterised by a tensile stiffness $E_t$ and a yield stress $\sigma _{\text {max}}$. To account for the inner-fibre slippage observed in the experiments, fibres were allowed a ductile response before failure, defined by an accumulated tensile plastic strain $\varepsilon ^p_f$. In compression, the tensile stiffness was extended to compression up to a buckling stress $\sigma _{bk}$, at which point significantly lower compressive stiffness $E_c$ was assigned. The buckling stress and compression stiffness are phenomenological variables, meant to capture the in-matrix behaviour of the fibres. Consequently, they should be calibrated by back analysis of experimental data.

**Fig. 6:**
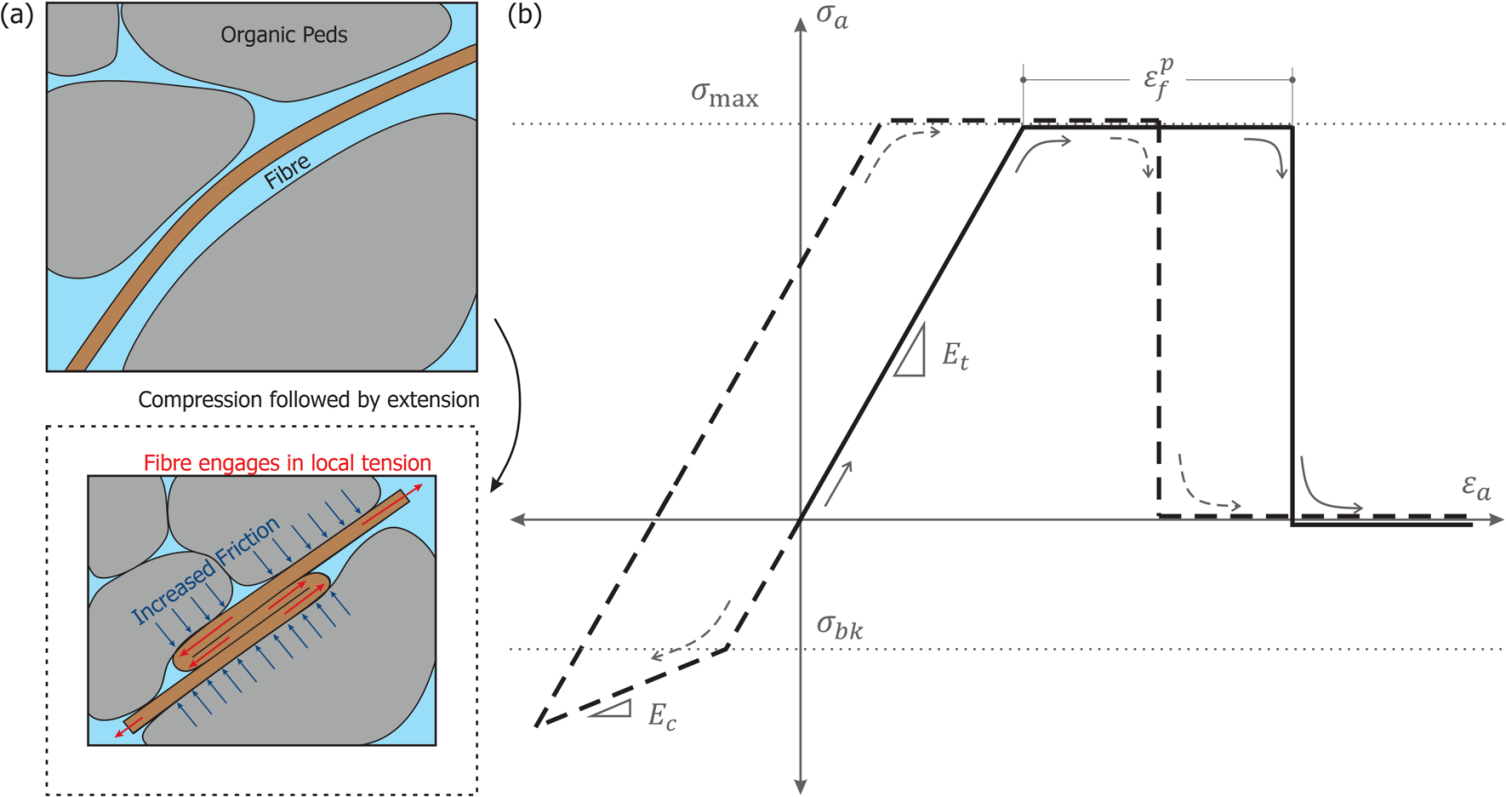
Conceptual fibre behaviour. (**a**) Mechanism for fibre engagement in tension after compression; (**b**) Fibre constitutive relationship: the solid line describes a fibre in monotonic tension, and dashed one experiencing strain reversal.

Special attention was given to the behaviour of fibres upon strain reversal. When compressed beyond their buckling stress, they fold and offer minimal resistance. However, upon subsequent extension, the now denser soil matrix increases friction and imposes constraints on the unfolding. As a result, the fibres re-engage in localised tension before returning to their original configuration. This mechanism is schematised in Fig. 6a. Under these considerations, the constitutive law prior to failure is conveniently expressed in incremental form, as summarised in Eq. (5). Figure 6b illustrates the resulting stress–strain behaviour.

4 $$\begin{aligned} \delta {\sigma }_a&= E_t (\delta {\varepsilon }_a - \delta {\varepsilon }_a^p) \end{aligned}$$

5 $$\begin{aligned} \delta {\varepsilon }_a^p&= {\left\{ \begin{array}{ll} \delta {\varepsilon }_a - (\sigma _{\text {max}} - \sigma _a^n) / E_t, & \text {if } \delta {\varepsilon }_a> 0 \quad \& \quad \sigma _a^e> \sigma _{\text {max}}, \\ (\delta {\varepsilon }_a - (\sigma _{bk} - \sigma _a^n) / E_t) (1 - E_c / E_t), & \text {if } \delta {\varepsilon }_a< 0 \quad \& \quad \sigma _a^e< \sigma _{bk} \quad \& \quad \sigma _a^n > \sigma _{bk}, \\ \delta {\varepsilon }_a (1 - E_c / E_t), & \text {if } \delta {\varepsilon }_a < 0 \quad \& \quad \sigma _a \ge \sigma _{bk}, \\ 0, & \text {else} \end{array}\right. } \end{aligned}$$

where $\sigma _a^e =\sigma _a + \delta {\varepsilon _a}E_t$ is an elastic trial and the superscript *n* in $\sigma _a^n$ denotes the stress at the end of the previous increment. Fibre breakage is introduced by tracking the accumulation of plastic strains in tension $\varepsilon ^{p+}_a$, according to Eq. (6), and fixing the axial stress of the fibre to be null if $\varepsilon ^{p+}_a \ge \varepsilon ^{p}_f$.

6 $$\begin{aligned} \delta {\varepsilon }^{p+}_a = {\left\{ \begin{array}{ll} \delta {\varepsilon }_a^p, & \text {if } \delta {\varepsilon }_a^p > 0 \\ 0 & \text {else } \\ \end{array}\right. } \end{aligned}$$

The stiffness and strength of each fibre were assigned by randomly sampling the experimentally derived Weibull distributions shown in Fig. 2b. For each fibre, its random diameter was used as the size metric in both distributions. Considering the non-linear stress-strain behaviour that was observed experimentally, a scaling factor $f_b$ was introduced in the formulation to reduce the stiffness of the fibres predicted by the Weibull distribution, such that $E_t = f_bE_w$. This factor is conceptually similar to the the scalar functions used to model other fibre reinforced soils^36–38^. The reduced stiffness attenuates the fibre contribution in a simplified manner accounting for fibre curliness while allowing the network to rotate as expected from the deformation gradient tensor. Additional details regarding its formulation can be found in the Methods section.

Given the stochastic nature of strength and stiffness, the maximum plastic strain before breakage, $\varepsilon ^{p}_f$, was defined as proportional to elastic strain at yield, such that $\varepsilon ^{p}_f = \alpha _d \cdot \sigma _{\text {max}} / E_t$, where $\alpha _d > 0$ is a ductility factor set to 1.0 in this study.

## Discussion

Numerical simulations with finite and infinitesimal strain formulations were compared to examine the influence of fibre kinematics, initial orientation and strain history on the mechanical response. For the sake of simplicity, buckling stress or compressive stiffness were not activated. Aiming to represent natural peat deposits the analysis was conducted using a vertically restricted fibre orientation distribution (Fig. 5d). Table 5 summarises the default properties used to model the fibres in this study. Definition of the stress-strain variables adopted is presented in the Methods section.

**Table 5:** Summary of fibre properties and their distributions.

Parameter	Description	Units	Distribution	Parameters
$\rho (\theta ,\phi )$	Orientation	−	Supershape	(Table 4 row 4)
$D_f$	Diameter	mm	Log-Normal	$\mu$: 0.15 ; *STD*: 0.10
*AR*	Aspect ratio	−	Deterministic	50.0
$E_w$	Tension stiffness	kPa	Weibull	(Table 2 row 4)
$\sigma _{\text {max}}$	Strength	kPa	Weibull	(Table 2 row 2)
$\alpha _d$	Ductility factor	−	Deterministic	1.0
$\sigma _{bk}$	Buckling stress	kPa	Deterministic	0.0
$E_c$	Compression stiffness	kPa	Deterministic	0.0
$f_b$	Stiffness scaling factor	−	Deterministic	1.0

### The importance of fibre kinematics

**Fig. 7:**
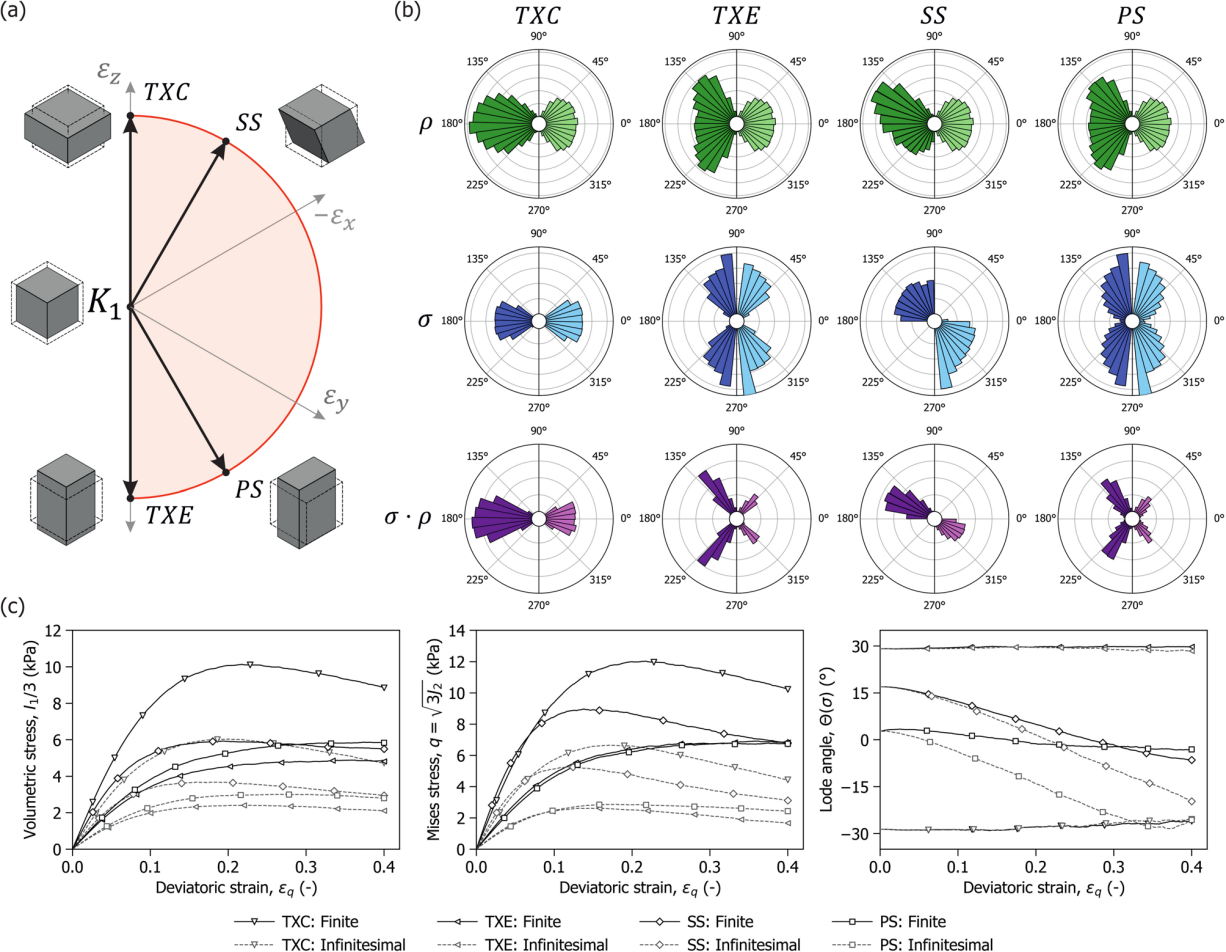
Influence of kinematics on fibre response. (**a**) Isochoric strain paths represented in the $\pi$-plane; (**b**) Polar plots showing the average fibre density (green), axial stress (blue), and reinforcement (purple) over the latitude angle. Dark and light tones correspond to finite and infinitesimal kinematics, respectively; (**c**) Evolution of the homogenised macroscopic fibre stress tensor invariants with deviatoric strain. From left to right: volumetric stress, Misses stress and Lode Angle.

The numerical sample was first subjected to isotropic compression to a volumetric strain $\varepsilon _v =0.4$, after which it was sheared under constant volume (isochorically) along four loading paths simulating typical undrained tests in soil mechanics: undrained triaxial compression (TXC), undrained triaxial extension (TXE), undrained simple shear (SS), and undrained pure shear (PS). Shearing continued until a deviatoric strain of $\varepsilon _q = 0.4$ was reached. Figure 7a illustrates the deformation mode associated with each shearing path and their directions in the strain $\pi$-plane. Note that TXC and TXE are shearing paths where compression and extension are applied respectively to the $z$ direction, with opposite strains in the $x$ and $y$ directions to maintain constant volume (i.e., undrained condition). SS and PS denote shearing paths with the same strain Lode angle ($\Theta (\varvec{\varepsilon }) = 0$) that differ in the rotation of the principal strain directions.

Figure 7b provides insight into the fibre response by showing polar plots of orientation density $\rho$, average axial stress $\sigma$, and resulting reinforcement $\sigma \rho$, each as a function of the latitude angle $\phi$. All quantities are evaluated at a deviatoric strain $\varepsilon _q = 0.2$. To facilitate comparison between the finite and infinitesimal approaches, half of the distribution of each scenario is shown on either side of the same polar diagram: finite strain on the left and infinitesimal strain on the right.

The first row of Fig. 7b illustrates the evolution of fibre orientation. In the infinitesimal case, the distribution remains fixed, whereas under finite strain kinematics, two effects emerge. Firstly, the area enclosed by the distribution increases due to the rise in volumetric fibre content caused by the initial compression. Secondly, as the sample is sheared, the distribution becomes increasingly aligned with the direction of major principal strain. The second row shows the average axial stress of the fibres along a given orientation. Stress differences between the two cases are less pronounced, although the finite strain scenario exhibits slightly higher values towards the major strain direction; this is attributable to fibre reorientation. The third row shows the overall directional reinforcement given by the product of the axial stress and the fibre density. Here, the influence of kinematics is most apparent with infinitesimal kinematics developing consistently lower reinforcement in all shearing paths and fibre directions.

Figure 7c presents the evolution of fibre reinforcement with deviatoric strain, quantified via the invariants of the homogenised fibre stress tensor. The results indicate that differences between the finite and infinitesimal strain formulations persist across the entire shearing process. They also highlight the role of loading direction in shaping the response of the fibre network. Strain paths such as TXC and SS, where tensile mobilisation aligns with the initial fibre orientation distribution, produce a stiffer initial response, associated with higher peak values of volumetric and von Mises stress. However, this response diminishes more rapidly at large strains due to fibre breakage reducing the proportion of fibres mobilised in tension. In contrast, the PS and TXE paths which impose tension in the vertical direction engage a smaller proportion of fibres of the vertically restricted distribution. This results in a more gradual reinforcement which is sustained by fibre rotation.

The evolution of the fibre stress Lode angle $\Theta (\varvec{\sigma })$, further clarifies the role of kinematic assumptions. In TXC and TXE, the axisymmetric configuration of both the fibre distribution and the loading path leads to minimal variation in the Lode angle, irrespective of the strain formulation. The effect of kinematics becomes more pronounced under non-axisymmetric loading. In SS, the finite and infinitesimal formulations yield similar results up to $\varepsilon _q \approx 0.1$, beyond which the finite strain response diverges as differences develop in the proportion and orientation of failed fibres. In PS, the absence of rotation in the principal strain directions leads to an earlier departure between the two formulations. In the infinitesimal case, the stress Lode angle decreases steadily; in contrast, under finite strain kinematics, the fibre network reorients with the loading, maintaining the stress Lode angle relatively constant. This evolution reflects the interaction between loading and the fibre orientation anisotropy. These observations emphasise the critical role of kinematic assumptions in capturing the response of fibre-reinforced materials under rotation of principal strains.

**Fig. 8:**
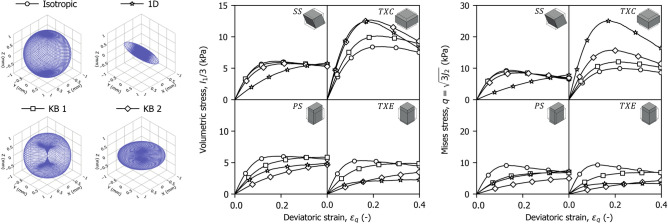
Influence of the initial fibre orientation on the fibre response for four isochoric shearing paths.

Naturally, fibre reinforcement depends on the initial orientation distribution and its alignment with the loading direction. This is illustrated in Fig. 8, where the shearing paths from Fig. 7 were applied to four supershape distributions: one isotropic, one one-dimensionally aligned with the *y* axis and two vertically restricted (KB1 and KB2) with increasing anisotropy.

The results show that reinforcement varies with the proportion of fibres mobilised in tension. In TXC, where tension develops horizontally, the one-dimensional distribution and KB2 provide the greatest reinforcement due to their larger proportion of horizontal fibres. This trend is reversed in TXE and PS, where vertical tension leads the isotropic distribution to mobilise the highest stress. Under SS, the responses of the axisymmetric distributions (i.e., isotropic, KB1, KB2) are similar. The one-dimensional distribution develops reinforcement qualitative similar to distributions with higher horizontal concentrations, however its stiffness both in SS and TXC reflects the lower and higher amount of fibres in the $x$ and $y$ direction respectively.

### The effects of compression strain history

**Fig. 9:**
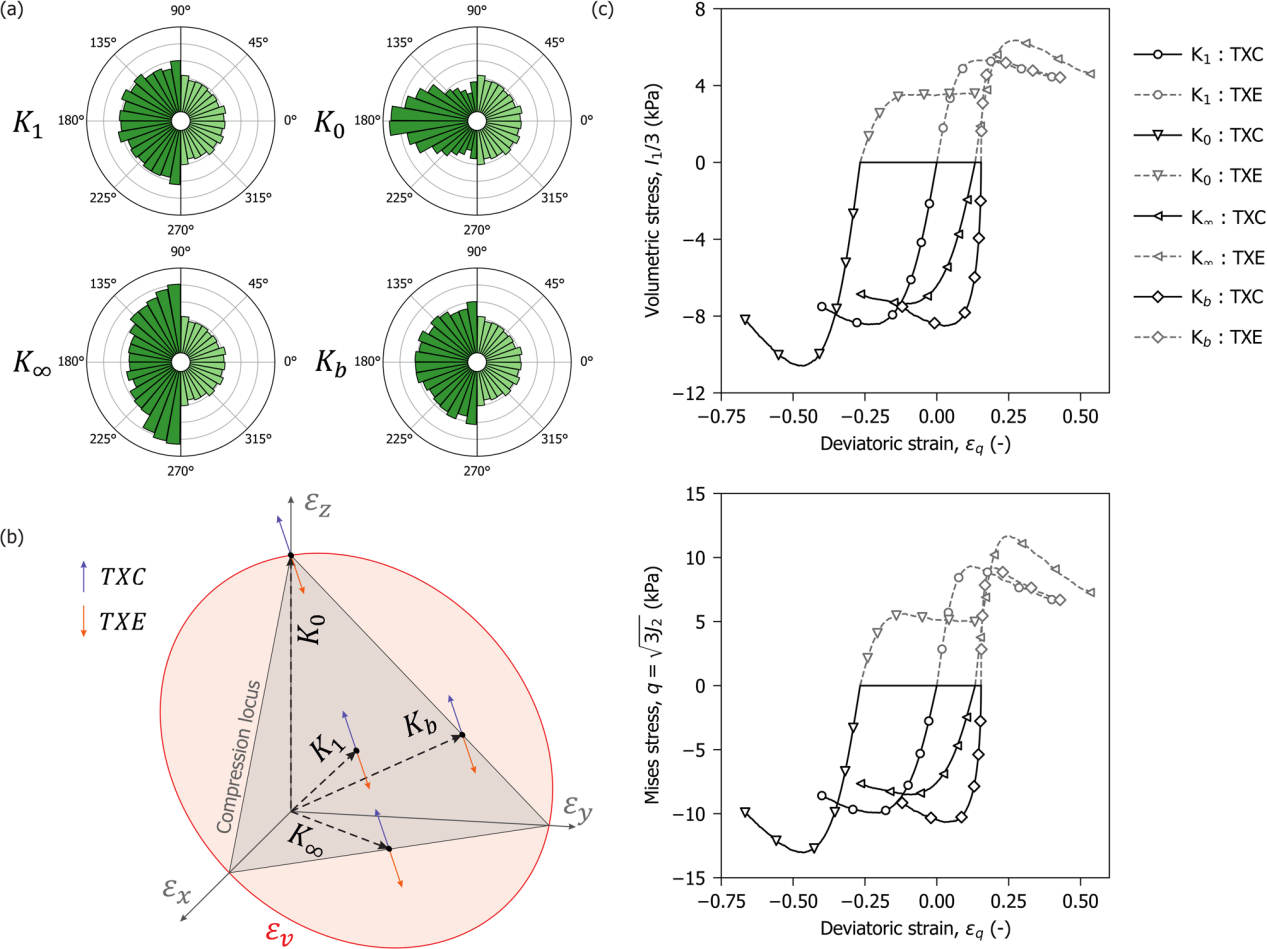
Consequences of compression strain history on peat shearing response. (**a**) Comparison of fibre orientation distributions after compression to the left, and initial to the right; (**b**) Compression paths in the principal strain domain; (**c**) Evolution of compression and deviatoric stress invariants with deviatoric strain.

The previous analysis highlighted the significant role of fibre reorientation, only upon shearing after isotropic compression. In practice, however, peat deposits often undergo compressive strain paths that induce reorientation prior to shearing. To isolate the effects of the prior strain history on the mechanical response, the simulations in this section assume an initial isotropic fibre orientation distribution that evolves through different compression paths before shearing. Four purely compressive paths, each reaching a final volumetric strain of 0.4 were imposed: isotropic compression ($K_1 \ni \varepsilon _x=\varepsilon _y=\varepsilon _z$); one-dimensional compression ($K_0 \ni \varepsilon _x,\varepsilon _y=0$); transverse plane compression ($K_\infty \ni \varepsilon _x =\varepsilon _y, \varepsilon _z=0$); and biaxial compression ($K_b \ni \varepsilon _x = 0, \varepsilon _y=2\varepsilon _z$). These paths are illustrated in the principal strain space in Fig. 9b, along with the resulting changes in the initially isotropic fibre orientation density (Fig. 9a).

Following compression, the samples were sheared isochorically along TXC and TXE paths. Figure 9c shows the evolution the volumetric and deviatoric invariants of the stress tensor with deviatoric strain, scaling each value by the sign of the corresponding third invariant $J_3$ to preserve a notion of loading orientation. The results demonstrate that the fibre response is strongly influenced by the alignment between the principal strain directions of compression and those of subsequent shear. When the directions coincide, as in $K_0$ followed by TXC or $K_\infty$ followed by TXE, fibre alignment is monotonic and results in enhanced reinforcement and higher peak stress invariants. In contrast, configurations involving directional reversal, such as $K_0$ followed by TXE or $K_\infty$ followed by TXC, exhibit weaker reinforcement due to fibres going back and forth between orientations.

A closer comparison between the $K_b$ and $K_1$ compression paths offers further insight. Although the loading histories differ, the fibre response during subsequent shearing remains remarkably similar. In both $K_b$ and $K_1$, the projection of the compression strain on the $\pi$-plane is oriented orthogonally to the shearing strain increments. This suggests that the reinforcement developed during shear is not determined solely by the characteristics of the compression stage, but rather by the degree of alignment between the principal directions of compression and those of shear.

### Comparison with experimental data

**Fig. 10:**
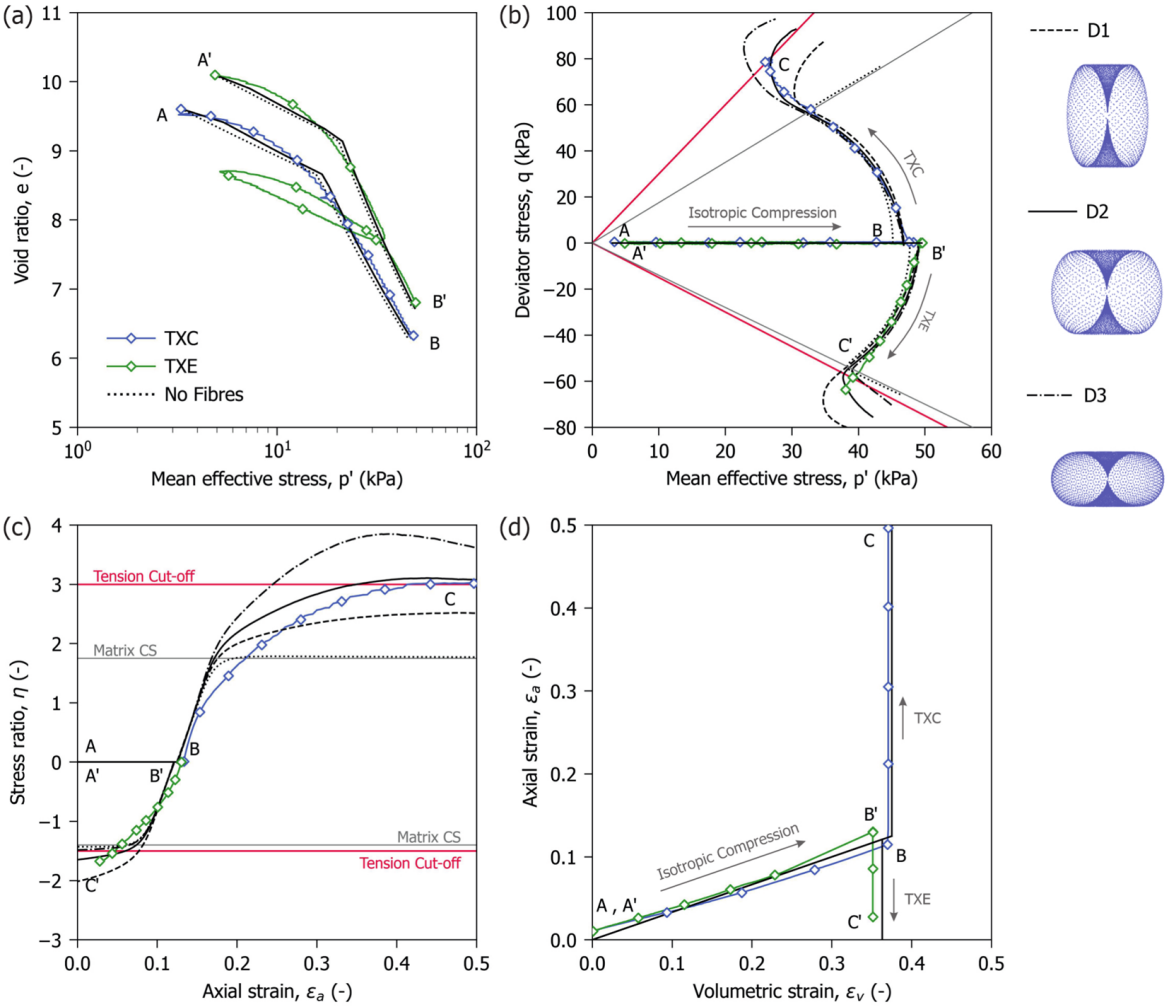
Comparison between triaxial tests on peat samples and the proposed numerical formulation. (**a**) Volumetric response during compression; (**b**) Stress paths of the samples; (**c**) Change of stress ratio of the samples with deviatoric strain; (**d**) Strain paths of the samples.

Validation of the proposed numerical framework was addressed by comparing its predictions with experimental tests. Two triaxial tests were performed using the equipment described in the Methods section. To minimise sample disturbance, cylindrical specimens (50 mm in diameter and 100 mm in height), were gently carved from a large cylindrical block sample of peat (400 mm in diameter and 450 mm in height) recovered from the Leendert de Boerspolder in the Netherlands. The samples were saturated under a back pressure of 300 kPa and isotropically compressed at a rate of 8 kPa/day to a mean effective stress of 50 kPa (A to B in Fig. 10). After compression, drainage was closed and the samples were sheared at constant volume in strain-controlled triaxial compression (TXC) and extension (TXE) at a rate of 0.015 mm/min (B to C).

Both tests were modelled using the calibrated statistical properties of the fibres described in-text reference to  Table 5 , with a scaling factor $f_b = 0.5$ applied to the fibre stiffness to account for their curl. Given the uncertainties regarding the initial fibre orientation density, three vertically restricted fibre orientation distributions inspired by the data of Kettridge and Binley (2008)^26^, and modified to have increasing levels of anisotropy were compared (D1, D2, and D3 as shown in Fig. 10). The buckling stress and compression stiffness were calibrated with reference to the compression curves shown in Fig. 10a. The matrix was represented using the calibrated elastic–plastic model proposed by Muraro and Jommi (2021)^12^. The model parameters are presented in the Methods section.

Figure 10 shows the comparison between the experimental data and the model predictions for the two tests. For reference results are plotted together with the response of a sample with pure matrix and no fibres. Figure 10a shows that the calibrated model predictions match the experimental data independent of fibre orientations. Figure 10b shows that during shearing the overall evolution of the sample stress path closely follows the experimental measurements up to the critical state stress ratio (CSL) of the matrix (dotted line with slope equal to $M_g$, shown in grey in Fig. 10b,c). Differences between the three distributions become apparent beyond this point, with the D2 distribution providing the best match to the experimental data in both compression and extension paths.

The simulations reproduce two key features of peat behaviour: the mobilisation of very high stress ratios, reaching the tension cut-off Line (continuous red line shown in Fig. 10b,c), and the characteristic change in the stress path curvature above the critical state stress ratio often reported by several authors^10,17,18,39^. Figure 10c shows that D2 has the most satisfactory agreement with the experimental data, although the model retains slightly higher stiffness beyond the critical state line compared to the experiments.

The numerical simulations indicate that, once the large strain kinematics is accounted for, the initial fibre orientation can be indirectly characterised through back-analysis of experimental data. Comparisons between strain paths under opposite loading directions are particularly informative, as they highlight the degree of anisotropy in the fibre alignment along specific orientations. It is worth mentioning that direct measurement of the initial orientation of peat fibres remains virtually unfeasible, as available techniques struggle to identify saturated fibres in natural conditions even when using specialised equipment and procedures. Moreover, these techniques are unfeasible in routine engineering applications. In this context, the proposed formulation could provide an alternative for indirect characterisation.

## Conclusions

This study examined the kinematics of peat fibres including their mechanical behaviour and reinforcement under large deformations. Given the experimental challenges associated with direct measurement of fibre kinematics, the analysis combined experimental characterisation with numerical modelling to investigate their role in the overall response.

The experimental campaign yielded one of the first systematic datasets on the geometric and mechanical properties of individual organic fibres in peat. Tensile tests revealed that fibre strength and stiffness are size-dependent and highly variable. Dynamic image analysis was used to quantify the size distribution of fibres from three different deposits, all of which exhibited log-normal diameter distributions across most of the fibre volume. These geometric and mechanical features were integrated into a unified stochastic framework, in which tensile properties were treated as random variables following the tenets of Griffith theory and fracture mechanics.

Numerical modelling demonstrated the importance of fibre kinematics in the development of fibre reinforcement, as quantified by the macroscopic fibre stress tensor. The increase in volumetric fibre content induced by compression, combined with the progressive realignment of fibres towards the direction of maximum strain, leads to enhanced magnitudes of both volumetric and von Mises fibre stress. Fibre reorientation influences not only the magnitude but also the evolution of stress anisotropy. This effect becomes especially relevant in non-axisymmetric configurations of strain or initial fibre orientation, and is most pronounced in loading paths involving rotation of the principal strain directions, such as simple shear.

Fibre contributions to the mechanical behaviour depend on their orientation relative to the loading direction. This anisotropy is consistent with previous observations of directional dependency in peat and underscores the importance of accurately capturing the initial fibre orientation in constitutive models. To this end, a flexible approach was introduced to describe the orientation distribution using a surface described by the spherical product of two supershapes. As fibre orientations evolve during deformation in a manner consistent with the imposed kinematics, this surface may serve as a state variable in future constitutive formulations.

The findings further highlight the relevance of compression strain history in shaping the subsequent contribution of fibres during shear. Reinforcement develops more effectively when the loading path preserves the alignment between the principal strain directions of compression and shear, and is diminished upon strain reversal. These results have direct implications for both modelling and experimental interpretation. The magnitude and anisotropy of fibre-induced reinforcement cannot be inferred from the current strain state alone, but rather emerge from the entire deformation history. Accurate modelling of peat behaviour under field conditions therefore requires constitutive frameworks that incorporate strain path dependency. Likewise, careful consideration must be given when interpreting laboratory measurements, as differences in compression history may lead to significantly different reinforcement, even under otherwise identical shearing conditions.

The numerical framework was compared with experimental data on the behaviour of peat at the centimetric scale. Simulations were shown to successfully reproduce several features of the mechanical behaviour of peat samples as observed from laboratory testing. Furthermore, by accounting for the large strain kinematics, calibration of model parameters to experimental data under different loading conditions can offer insights into the initial orientation of fibres within the material.

The proposed framework provides a versatile tool for investigating the mechanical contribution of fibres under finite deformations, while maintaining moderate computational cost. The model has been derived and calibrated from data at the centimetric scale and could be used to analyse field-scale problems. With additional numerical development, it could be integrated as a constitutive law at Gauss points within a finite-element mesh. Future work should examine the fibre–matrix interaction in greater detail and explore possible model reductions when fibre network geometry can be simplified.

## Methods

### Micromechanical tensile tests

#### Experimental setup

**Fig. 11:**
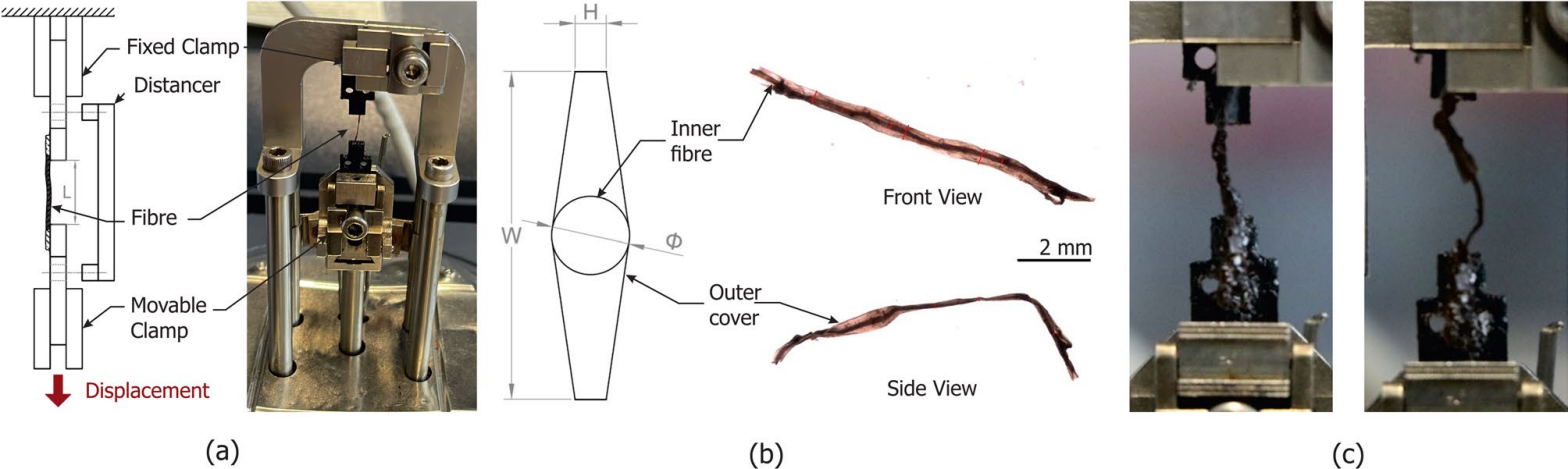
Tensile testing of peat fibres. (**a**) Testing setup; (**b**) Idealised cross-section used for as cross-sectional area and microscope observations of fibre 08B; (**c**) Inner fibre slippage observed in sample 20B.

Twenty-five peat fibres with diameters ranging from 0.25 mm to 2.58 mm were manually isolated from the soil specimen and stored in demineralised water, taking great care to minimise breakage or damage. Each fibre was affixed to a pair of polyethylene plastic clamps using cyanoacrylate adhesive and mounted on a TA Instruments Q800 dynamic mechanical analyser (DMA), capable of applying displacements and forces with resolutions of 1 nm and $1 \times 10^{-5}$ N, respectively (Fig. 11a). Twenty-three fibres were tested at a gauge length of approximately 8 mm, and two additional tests were conducted with lengths of 3 mm and 11 mm. The samples were pre-stretched under a force of $1 \times 10^{-4}$ N, their gauge lengths were recorded, and they were subsequently extended from the bottom at a constant rate of 200 $\mu$m/min until failure.

As natural materials, peat fibres exhibit significant variability in cross-sectional area due to changes along their length, damage, and other inhomogeneities. To address this, microscope measurements of postmortem specimens were used to determine an average or representative cross-sectional area for each fibre, from which an equivalent circular diameter was calculated. Initial observations revealed that most fibres are composed of a relatively flat outer layer surrounding a cylindrical inner fibre. Consequently, the cross-sectional area was modelled as an oblong hexagon, as shown in Fig. 11b. Two orthogonal microscope views were taken to assess the different dimensions (Fig. 11c). The test was interpreted using the Cauchy stress and logarithmic strains with a cross-sectional area given by Eq. (7).

7 $$\begin{aligned} A = \frac{1}{2} (H +\Phi ) * W \end{aligned}$$

All tests concluded with a sudden brittle fracture, accompanied by rapid displacement of the testing actuator. Accordingly, stress and strain calculations were performed assuming a constant cross-sectional area. In several cases, failure occurred when the inner fibre observed under the microscope slipped out of the outer covering. This phenomenon was particularly evident in Test 20B, where the inner fibre remained attached to the testing plates after the outer shell failed (Fig. 11b). Subsequent testing of the remaining inner fibre (Test 20B*) exhibited a linear stress–strain response with higher stiffness and strength. The slippage provided a mechanism for apparent ductility that could not be captured by the active load and strain control of the equipment.

**Fig. 12:**
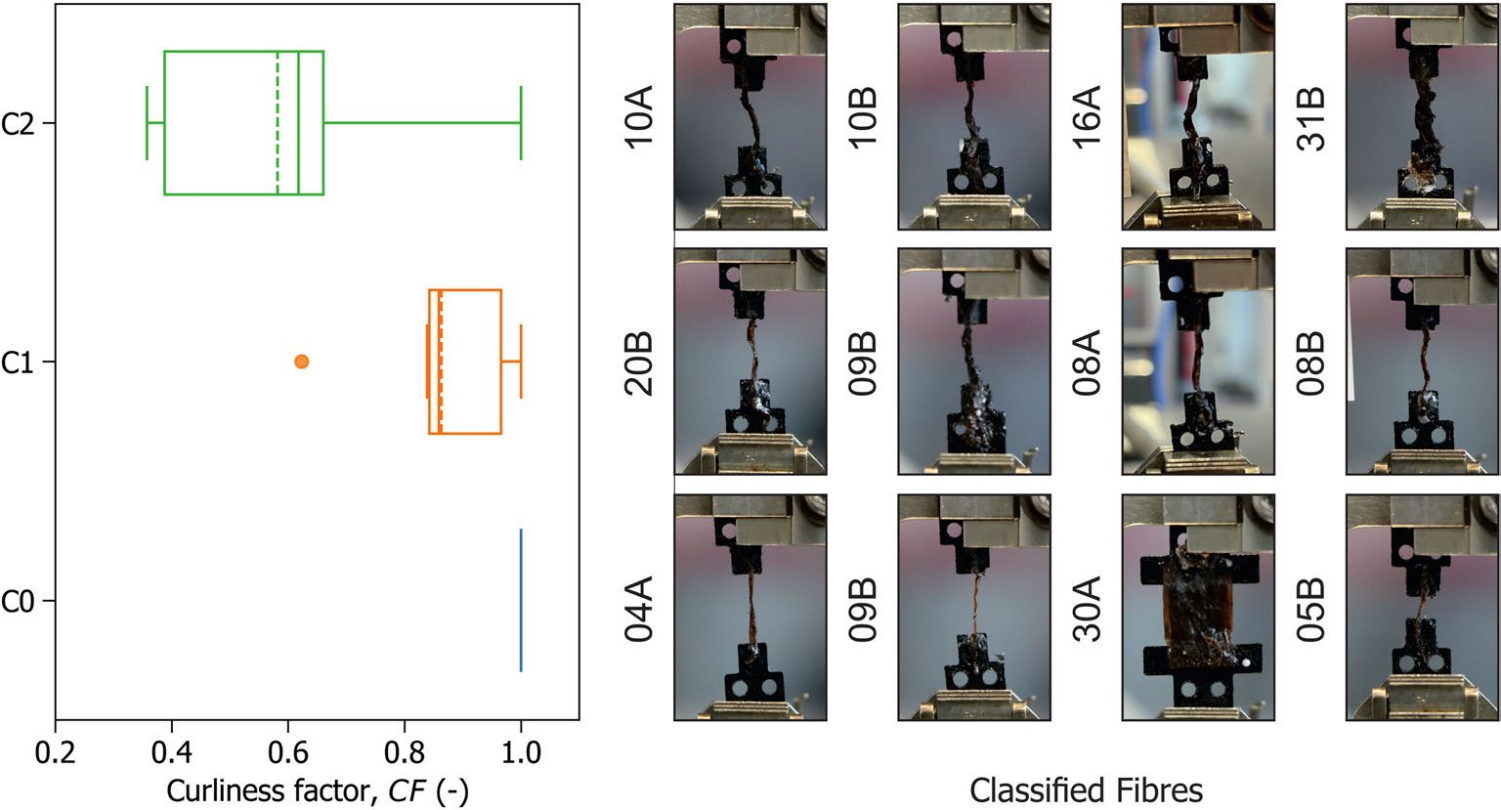
Curliness factor CF for different fibre categories.

Some tests exhibited progressive stiffening of the fibre as the fibre was straightened, which was associated with fibre curliness. Based on photographic records of the tests, a visual classification of fibre curliness in three categories (C0, C1, C2) was conducted to explore its relationship to the observed non-linear response. Fibres were photographed after being subjected to a minimal preload of approximately 0.1 mN. Classification was performed qualitatively, by comparing the fibre profile to the straight line joining its attachment points and assessing the degree of apparent deviation from the line. Three categories were defined: C0 for fibres where no significant curls were visible before tension, C1 for fibres where moderate undulations could be distinguished although the fibre appeared generally straight, and C2 for fibres showing pronounced curling. This procedure was based on two-dimensional images and represents a qualitative, first-order categorisation intended to capture differences in initial geometry. As a reference, examples of the classified fibres are shown in Fig. 12.

A quantitative measure of the non-linear behaviour was constructed by comparison of two stiffnesses. One measured by manually selecting for each test the linear region were the fibre is fully straightened and deriving its slope ($E_t$), and one describing the average slope of the whole stress-strain curve fitted by simple linear regression ($E_{reg}$). A curliness factor *CF* was defined as the ratio between the two slopes ($CF=E_{reg}/E_t$) with values of 1 denoting a linear response with constant stiffness, and values below 1 denoting convexity. A box plot for with respect to the visual categories is shown in Fig. 12.

Figure 12 indicates that the observed constitutive response is convex due to the fibre being gradually straightened as it is pulled.

#### Statistical interpretation

For single fibres, the maximum stress they can withstand is governed by flaws or weaknesses at discrete points rather than by the intrinsic material strength. As a result, fibre resistance follows a statistical distribution that reflects the probability of encountering a flaw with a strength equal to the applied stress. Within the framework of fracture mechanics and Griffith theory^40^, it is common to model the probability of failure of a fibre using a three-parameter Weibull distribution of the form:

8 $$\begin{aligned} P_f = 1 - \exp \left[ - \left( \frac{\sigma - \sigma _t}{\sigma ^*}\right) ^m \right] \end{aligned}$$

where $P_f$ denotes the probability of failure at an axial stress $\sigma$, and $\sigma ^*$, $\sigma _t$, and $m$ are the scale, location, and shape parameters of the distribution, respectively. The shape parameter $m$ governs the rate at which the failure probability increases with stress level, and the location parameter $\sigma _t$ can be interpreted as the minimum strength required for sample preparation^41^. The scale parameter $\sigma ^*$ represents the characteristic strength of the fibres and is often adapted to account for the dependence of failure probability on fibre size. For peat fibres, the characteristic strength was modelled according to:

9 $$\begin{aligned} \sigma ^* = \sigma _0 \left( \frac{V_0}{V_f}\right) ^{\gamma _s} + \sigma _m \end{aligned}$$

where $\sigma _0$ is the characteristic strength of a fibre of reference size $V_0$, $V_f$ is the size of the fibre, and $\gamma _s$ is a scaling parameter. The first term derives from the commonly applied principle of weak-link scaling^27^, where $\gamma _s$ modulates the increase in failure probability with size. The constant term $\sigma _m$ was introduced in this study to bound the reduction in strength with size. This accounts for the reduced strength degradation observed in larger fibres, which decompose more slowly due to their smaller ratio of surface area to volume.

The resulting distribution predicts the average fibre strength as follows:

10 $$\begin{aligned} \mathbb {E}\left( \sigma \right) = \left[ \sigma _0 \left( \frac{V_0}{V}\right) ^{\gamma _s} + \sigma _m \right] \Gamma \left( 1+ \frac{1}{m}\right) + \sigma _t \end{aligned}$$

where $\Gamma$ represents the gamma function. The associated probability density is given by:

11 $$\begin{aligned} \frac{\partial P_f}{\partial \sigma } = \frac{m}{\sigma ^*} \left( \frac{\sigma - \sigma _t}{\sigma ^*} \right) ^{m-1} \exp \left[ -\left( \frac{\sigma -\sigma _t}{\sigma ^*}\right) ^m\right] \end{aligned}$$

This statistical framework can be extended to account for the size dependence of the tensile stiffness $E_t$. In this case, $P_f$ represents the conditional probability of a fibre exhibiting an apparent stiffness lower than a given threshold $\sigma$. A probabilistic analogy with strength is justified by considering that the apparent stiffness may be influenced by varying levels of decomposition and reductions in cross-sectional area.

### Dynamic image analysis

**Figure 13:**
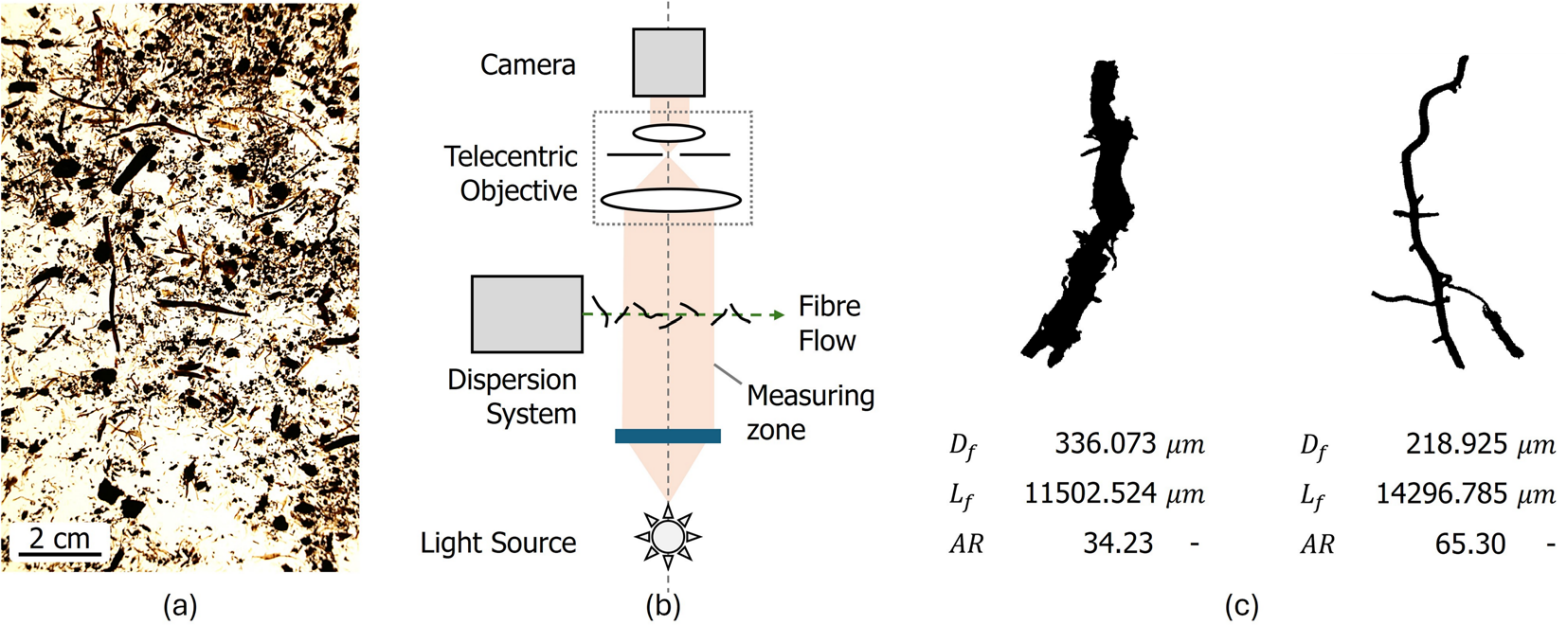
Working principle and examples of dynamic image analysis measurements. (**a**) Water dispersed fibres, (**b**) DIA working principle, (**c**) Example of two measurement results.

Size and shape measurements of fibres from three different peat deposits in the Netherlands were conducted using dynamic image analysis (DIA) with a QicPic system and FlowCell module from Sympatec GmbH^42^. For each deposit, two to five cubic peat samples of approximately 200 g were tested. The QicPic system utilises a telecentric optical arrangement to capture high-speed images of soil particles as they flow, dispersed in a carrier medium, through the measurement zone. These images are subsequently processed to derive various shape parameters based on the projected area of the particles. Figure 13b illustrates the working principle of the DIA system, and Fig. 13c provides an example of the data obtained. In this study, each sample contained, on average, 250,000 particles, resulting in up to 1.2 million fibre measurements per deposit. Figure 13a shows an example of the tested fibres.

The fibres were isolated by disaggregating the peat in a 5% molar solution of sodium hexametaphosphate, then separated through sedimentation and wet sieving using a 450 $\upmu \hbox {m}$ mesh. After isolation, small quantities of fibres were dispersed in water and placed into a raised tank. The fibres were circulated by gravity through the optical system and collected in a lower receiving container. Samples were dispersed and circulated sequentially until more than 150,000 fibres per sample had been observed.

The telecentric objective and measurement zone were configured with a pixel resolution corresponding to a minimum identifiable particle size of 17 $\upmu \hbox {m}$, and a maximum measurable dimension of 33792 $\upmu \hbox {m}$. To ensure a meaningful characterisation, shape analysis was conducted only for particles larger than 153 $\upmu \hbox {m}$; particles with lengths below this threshold were excluded from the analysis.

Image analysis enables the derivation of a wide range of shape descriptors for each particle; however, this study focused on the average fibre length and diameter. Fibre shape identification was achieved by computing the topological skeleton of each observed fibre image using a skeletonisation algorithm^43^. The average fibre diameter, $D_f$, was determined by taking the ratio of the total pixel area to the total length of the skeleton, while the fibre length, $L_f$, was defined as the length of its longest branch. To complement these measurements, a simple complexity index $C$ was recorded as the inverse of the total number of paths within the skeleton. The volume of each fibre was approximated as that of a cylinder, with diameter and height corresponding to $D_f$ and the total length of the skeleton, respectively.

DIA enables the shape characterisation of a statistically significant number of fibres across a wide range of potential shapes and sizes. However, the procedure requires isolating the fibres from the peat matrix, which introduces biases related to fibre breakage during sample preparation. Despite careful handling, some fibre dimensions were inevitably reduced due to longitudinal breakage or the loss of outer layers. The statistical distribution of fibre length was likely affected disproportionately. In addition, for the tested samples, the measuring range of the equipment prevented the detection of fibres with diameters greater than 2 mm.

### Stress strain variables

The invariants of a second-order tensor $\textbf{T}$, such as the stress tensor $\varvec{\sigma }$ or the strain tensor $\varvec{\varepsilon }$, are are defined as:

12 $$\begin{aligned} I_1 = \textrm{tr}(\textbf{T}), \qquad I_2 = \frac{1}{2}\left[ (\textrm{tr}\textbf{T})^2 - \textrm{tr}(\textbf{T}^2)\right] , \qquad I_3 = \det (\textbf{T}), \end{aligned}$$

Here, $I_1$ represents the volumetric contribution and is used to define the volumetric strain $\varepsilon _v = I_1$, and the volumetric stress $p = I_1/3$, which is scaled to average out the contribution of the three directions. The deviatoric part of the tensor is defined as $\textbf{s} = \textbf{T} - \frac{1}{3} I_1 \textbf{I}$, where $\textbf{I}$ is the identity tensor. Its invariants are

13 $$\begin{aligned} J_1 = \textrm{tr}(\textbf{s}) = 0, \qquad J_2 = \frac{1}{2}\textrm{tr}(\textbf{s}^2), \qquad J_3 = \det (\textbf{s}), \end{aligned}$$

with $J_1$ vanishing by construction. The second deviatoric invariant $J_2$ of the stress tensor $\varvec{\sigma }$ is commonly used to quantify shear intensity by means of the Misses stress $q = \sqrt{3J_2(\varvec{\sigma })}$, wich is a work conjugate to the deviatoric strain $\varepsilon _q = \sqrt{4J_2(\varvec{\varepsilon })}$. The Lode angle $\Theta$ for either tensor is defined in this work as:

14 $$\begin{aligned} \sin (3\Theta )=\frac{J_3}{2} \cdot \left( \frac{3}{J_2}\right) ^{3/2} \end{aligned}$$

The use of $\varvec{\sigma }$ and $\varvec{\varepsilon }$ is adopted to represent the stress and strains of the composite, while the subscripted $\varvec{\sigma _m}$ and $\varvec{\varepsilon _m}$, and $\varvec{\sigma _f}$ and $\varvec{\varepsilon _f}$ are used to indicate the tensors of the matrix and fibres respectively.

The experimental data was elaborated adopting common triaxial stress variables, namely the mean effective stress $p'$, and the deviatoric stress *q*. The mean effective stress, conventionally used in soil mechanics, is the first invariant of the effective stress tensor $\varvec{\sigma '}$ given by:

15 $$\begin{aligned} \varvec{\sigma '} = \varvec{\sigma } - \textbf{I}u \end{aligned}$$

were *u* denotes the magnitude of the pore water pressure. Note that in the simulations, no coupling to pore pressure is introduced thus the stress tensors should be regarded as effective.

### Random generation of fibre distributions

The fibre distributions can be realised from the fibre orientation function using a discretisation-based sampling method. This approach approximates the continuous distribution $\rho (\theta , \phi )$ by sampling the function evenly on the unit sphere and enables fibre generation according to its values. The general workflow to generate a virtual sample with a target fibre content $\hat{\mu }_f$ and the desired fibre distribution $\rho (\theta , \phi )$ is as follows:

1. The orientation distribution function $\rho (\theta , \phi )$ is sampled evenly on the unit sphere using a spherical design^44^. In this work the symmetrical spherical design of order 141 with 10014 nodes found by Womersely (2018)^45^ was used. Let $\{\theta _i\}_{i=1}^{N_\theta }$ and $\{\phi _j\}_{j=1}^{N_\phi }$ represent the discrete points of the quadrature on the $\theta$- and $\phi$-axes, respectively. For each pair $(\theta _i, \phi _j)$, the value $\rho (\theta _i, \phi _j)$ is computed.
2. A list $\mathcal {L}$ is created where each pair $(\theta _i, \phi _j)$ is represented by an integer number of occurrences proportional to $\rho (\theta _i, \phi _j)$. Specifically, the pair $(\theta _i, \phi _j)$ is replicated $\lfloor k \cdot \rho (\theta _i, \phi _j) \rfloor$ times in $\mathcal {L}$, where $k$ is a scaling factor chosen to ensure that all values are integers and the list size is manageable. This step effectively creates a discrete approximation of the continuous density function by converting $\rho (\theta , \phi )$ into a weighted list that reflects the relative probabilities.
3. A cartesian volume *V* is defined by specifying finite ranges for the three spatial directions (*x*, *y*, *z*) as defined in equation (16). In this study the domain was set to be cubic with a size equal to 50 mm. The size was chosen to ensure the existence of a representative elementary volume (REV) and to keep the simulations comparable to the scale of laboratory samples.
  16 $$\begin{aligned} V = \{ (x, y, z) \in \mathbb {R}^3 \mid x_a \le x \le x_b, \; y_a \le y \le y_b, \; z_a \le z \le z_b \} \end{aligned}$$
4. A random coordinate $M_i = (x_i, y_i, z_i)$ within *V* is selected assuming an uniform spatial distribution. The point $M_i$ defines the centre point of fibre *i*.
5. A fibre orientation is selected for the fibre by sampling $\theta$ and $\phi$ randomly from the $\mathcal {L}$ list.
6. A length diameter $D_i$ for the fibre is selected by sampling from the corresponding probability density $P_D$. In this study, the length $L_i$ was assigned according to a deterministic aspect ratio.
7. Start and end points of the fibres are defined based on the length and orientation $\xi _i$, according to:
  17 $$\begin{aligned} \varvec{\xi }_i = \left( \cos \phi _i \cos \theta _i, \; \cos \phi _i \sin \theta _i, \; \sin \phi _i \right) ; \quad S_{i} = M_i - \varvec{\xi }_i \frac{L_i}{2} \; ; \quad E_{i} = M_i + \varvec{\xi }_i \frac{L_i}{2} \end{aligned}$$
8. Other physical or mechanical properties of the fibre are assigned by sampling from their respective distributions (e.g. fibre stiffness, fibre strength) conditionally to the fibre size.
9. The fibre volume is calculated as $v_i = L_i D_i^2 \pi / 4$, and steps 4 to 8 are repeated until the total fibre content is equal or greater to the target, that is:
  18 $$\begin{aligned} \frac{1}{V} \sum _{i=1}^{n} v_i \ge \hat{\mu }_f \end{aligned}$$

Note that fibre generation targets a volumetric proportion of fibres $\mu _f$ within the total sample volume *V*. Direct experimental measurement of the volumetric fibre content $\mu _f$ is often challenging or impractical. However, the volumetric proportion can be computed as a function of the gravimetric fibre content *FC*, which is easily measurable in the laboratory. The volumetric and gravimetric counterparts are related through the specific gravity of the fibre solids ($Gs_f$), the overall specific gravity of the sample solids (*Gs*), and the fibre and overall sample porosities ($n_f$ and *n*), as given in equation (19).

19 $$\begin{aligned} \mu _f = FC \cdot \frac{Gs}{Gs_f} \cdot \frac{1-n}{1-n_f}. \end{aligned}$$

#### On the distinction between matrix and fibres

In principle, defining a cutoff size between fibres that act as kinematic restraints and those that form the organic matrix is inherently challenging due to the continuous, fractal-like nature of the fibre arrangement, shown in the ESEM micrographs of Figure 1.

Peat fibre content is measured following standards such as the ASTM D1997, which imposes a separation through wet sieving. The DIA measurements presented in Fig. 3 show that 100 $\upmu \hbox {m}$ lies at the lower end of the diameter distribution when fibres are isolated by following similar procedures. Additionally, previous studies^12^ distinguished the diffuse fibres of a reconstituted peat matrix without large fibres at a length of approximately 3 mm (i.e., a diameter between 50 and 200 $\upmu \hbox {m}$, based on aspect ratios from our tensile tests). The constitutive model they proposed was demonstrated to be suitable for describing the organic matrix of natural peat^20^, but also to require additional description to handle larger fibres.

Given these considerations, in this work a threshold of 100 $\upmu \hbox {m}$ was chosen to differentiate between the organic matrix and the reinforcing fibres. The diameter distributions used for sample generation were truncated accordingly. Notably, this choice also naturally implies an upper limit to the stiffness and strength values predicted by the Weibull distributions used to model the mechanical properties.

### Considerations for non-straight fibres

**Fig. 14:**
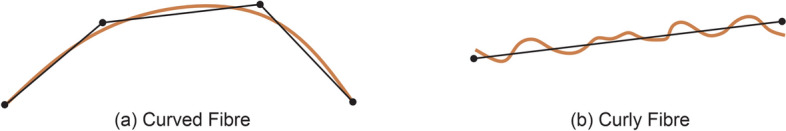
Types of non straight fibres.

The proposed model uses perfectly straight fibres as a simplification of the complex arrangement of natural peat fibres. However, the effects of two non-exclusive types of curvature could be considered: “curved fibres” with a radius of curvature comparable to the fibre length, and “curly fibres” having a main orientation with multiple curves with a radius of curvature considerably smaller than the fibre length. These are schematized in Fig. 14.

Under the model assumptions, where displacements are considered homogeneous and fibre connectivity is neglected, curved fibres can be reasonably approximated by a series of smaller straight segments. This is because each segment may engage in a localised tensile response due to the restrictions imposed by the presence of the matrix^46^, as shown in Fig. 6. Within the stochastic nature of the model, stresses of randomly located and oriented fibres are homogenised through a linear composition. Consequently, the response of a set of long curved fibres would be equivalent to a set of shorter straight fibres, provided the latter describes the same volume fraction. In other words, under the model assumptions, we can describe the influence of a long-curved fibre as a sum of shorter straight segments.

It is worth noting that in practice, it is not trivial to define an orientation for a curved fibre, and doing so requires an assumption on the scale of elementary elements. For example, Kettridge and Binley (2008), measured fibre orientations by considering stems and branches as approximately straight elementary units, which is consistent with the segment-wise model. The way fibres are generated must be consistent with the way that orientation densities are derived.

Curly fibres do have a general orientation but exhibit a progressive increase in stiffness as they engage in tension, as observed in the non-linear responses measured in the tensile tests. However, it would be inaccurate to directly apply the same non-linear response measured in the tests to fibres within peat. The non-linearities observed in the tensile tests are partially related to the initial curliness of the fibres as set up in the experiment. This initial curliness results from the fibre constitution, and the procedure followed to separate the fibres from the matrix and attach them to the clamps. Consequently, the effective curliness and the magnitude of the non-linear behaviour observed are dependent on the experimental setup. Furthermore, during the test the fibre is subjected to free elongation in the absence of any restraints from the surrounding matrix.

A way to consider the effects of fibre curliness is the use of an interaction factor, $f_b$, mediating the transmission of strains from the composite to the fibre axial direction, as proposed for other soils^36–38^. This effect is introduced in this formulation as a scaling factor to the tensile stiffness predicted by the Weibull distribution. This attenuates the fibre contribution in a simplified manner which accounts for of the curliness while allowing the network to rotate as expected from the deformation gradient tensor.

### Finite strain implementation

Following the principles of finite strain theory^47^, the composite sample undergoes a continuous motion $\chi (\textbf{X}, t)$, which maps the material coordinate $\textbf{X}$ of a solid point to its corresponding coordinate in the deformed configuration $\textbf{x}$ at time *t*. Under the assumption that the motion is homogeneous, the deformation can be described by a linear transformation entirely defined by the deformation gradient tensor $\textbf{F}$. The logarithmic strain of the sample can be derived from the polar decomposition of $\textbf{F}$ into a rotation tensor $\textbf{R}$ and a stretch tensor $\textbf{U}$, as expressed in Eq. (20):

20 $$\begin{aligned} F_{i,j} = \frac{\partial \chi _i}{\partial X_j}\ \ \ ;\ \ \ \textbf{F} = \textbf{R}\textbf{U}\ \ \ ;\ \ \ \mathbf {\varepsilon } = \ln \textbf{U} \end{aligned}$$

Fibres are embedded within the composite and must deform in accordance with the motion of the composite. While slippage between the fibres and the matrix may occur, the fibres are still expected to rotate in response to the distortional strains of the composite. Disregarding slippage initially and assuming the fibres remain straight, it is sufficient to apply the motion $\chi (t)$ to the initial start and end coordinates ($\textbf{S}_0$ and $\textbf{E}_0$) of the fibres to determine their deformed configuration. Consequently, each deformed fibre can be represented by a vector, $\varvec{\xi }_t$, determined by the deformation gradient tensor and the initial fibre configuration, $\mathbf {\xi }_0$, as shown in Eq. (21).

21 $$\begin{aligned} \varvec{\xi }_t = \textbf{E}_t - \textbf{S}_t = \textbf{F}(t) \varvec{\xi }_0 \end{aligned}$$

Upon deformation, each individual fibre experiences an axial logarithmic strain equal to:

22 $$\begin{aligned} \varepsilon _a = \ln {\left( \frac{\Vert \varvec{\xi }_t\Vert }{\Vert \varvec{\xi }_0\Vert }\right) } \end{aligned}$$

The strain increment in each fibre can be used to compute the corresponding axial stress $\sigma _a$ by considering the cross-sectional area $A_i$ and applying an appropriate constitutive relation. The resulting internal forces for the *n* fibres are then homogenised into a single stress tensor, $\varvec{\sigma _f}$, representative of the mechanical state of the fibres, using the Gauss-Ostrogradsky theorem. The tensor is given by Eq. (23),

23 $$\begin{aligned} \varvec{\sigma _f} = \frac{1}{V(t)}\sum ^n_{i=1} \frac{\sigma _a^i(t) A_i}{L_i(t)} \left( \varvec{\xi }_t \otimes \varvec{\xi }_t \right) \end{aligned}$$

where $\otimes$ denotes the dyadic product, $L_i(t) = \Vert \varvec{\xi }_t\Vert$ is the current length of the fibre, and *V*(*t*) is the current volume of the sample.

Note that the model represents the fibre network as tensional kinematic constraints superimposed to the effects of the matrix. The overall stress response of the composite $\varvec{\sigma }$ is given by the sum of the homogenised fibre stress $\varvec{\sigma _f}$, and the stress obtained by integration of the constitutive relation of the matrix $\varvec{\sigma _m}$ as:

24 $$\begin{aligned} \varvec{\sigma } = \varvec{\sigma _m} + \varvec{\sigma _f} \end{aligned}$$

With the strains defined as,

25 $$\begin{aligned} \varvec{\varepsilon } = \varvec{\varepsilon _m} = \varvec{\varepsilon _f} \end{aligned}$$

The assumption of equal strains in all constituents is motivated by the ESEM micrographs of Fig. 1. It can be seen that peat fibres form a tangled network with high coordination numbers and branching, facilitating force transmission between the fibres and the organic matrix. The matrix itself exhibits a non-linear and non-reversible behaviour with strain hardening upon compression. The relatively compliant fibres will bond more effectively with increasing confining stress. While fibre slippage has been observed at the onset of non continuous displacement fields^48^, this condition lies beyond the scope of the model which focuses on the pre-failure response where the material remains continuous.

### Infinitesimal strain computations

For the infinitesimal case, fibre strains were calculated by projection of the sample strain tensor $\varvec{\varepsilon }$ to the original fibre orientation vector $\varvec{\xi _0}$ such that,

26 $$\begin{aligned} \varepsilon _a =\frac{1}{\Vert \varvec{\xi _0}\Vert ^2} \varvec{\xi _0}^T \varvec{\varepsilon }\varvec{\xi _0} \end{aligned}$$

Subsequent stress derivations are conducted as for the finite strain case.

### Triaxial tests

The tests were carried out using a load frame triaxial apparatus with back pressure and cell pressure volume controllers and submersible 1 kN load cell (± 1 N), under controlled room temperature 14±1 $^{\circ }\hbox {C}$. The accuracy of the controllers was ±1 kPa on pressure and ±200$\hbox {mm}^{3}$ on volume (both at 0.1% full scale range, FSR). Thin membranes 0.25 mm thick were used. To accelerate the consolidation process lateral filter paper was placed around the samples. Additional information regarding the apparatus and testing procedure is described by Lade^49^ and Muraro & Jommi^19^.

### Modelling of peat matrix

For the experimental comparison the matrix was described using the calibrated parameters of the model proposed by Muraro & Jommi (2021)^12,20^ generalised to multiaxial conditions^50^. This models features modifications with respect to the widely used MCC model^51^. The yield locus is defined as:

27 $$\begin{aligned} f= q^2+ \frac{ M_{\textrm{f}}^2}{1-\chi _{\textrm{f}}} \left( \frac{p^{\prime }}{p_{\textrm{c}}^{\prime }}\right) ^{\frac{2}{\chi _{\textrm{f}}}} p_{\textrm{c}}^{\prime 2}- \frac{ M_{\textrm{f}}^2 p^{\prime 2}}{1-\chi _{\textrm{f}}} \end{aligned}$$

where $p'_c$ is the hardening variable. The plastic potential is described by a non-associated flow rule with a modified dilatancy relation *d*, which follows:

28 $$\begin{aligned} d=\frac{\delta \varepsilon _{\textrm{v}}^{\textrm{p}}}{\delta \varepsilon _{\textrm{q}}^{\textrm{p}}}=\frac{\beta _g^2 M_{\textrm{g}}^2-\eta ^2}{\chi _{\textrm{g}} \eta } \end{aligned}$$

where $\delta \varepsilon _{\textrm{v}}^{\textrm{p}}$ and $\delta \varepsilon _{\textrm{q}}^{\textrm{p}}$ are the increment of volumetric and deviatoric plastic strains respectively, and $\beta _g$ is equal to 1.0 in triaxial compression and 0.8 in triaxial extension. The model considers a mixed volumetric and distortional hardening rule defined as:

29 $$\begin{aligned} \frac{\delta p_{\textrm{c}}^{\prime }}{p_{\textrm{c}}^{\prime }}&= \frac{1}{\lambda ^*-\kappa ^*}\left( \delta \varepsilon _{\textrm{v}}^{\textrm{p}}+\left( D_0 \exp \left[ -D_1 \varepsilon _q^p\right] \right) \delta \varepsilon _{\textrm{q}}^{\textrm{p}}\right) \end{aligned}$$

The parameters employed in the model are reported in Table 6. The model was integrated following the algorithm proposed by Bardet & Choucair^52^.

**Table 6:** Summary of peat matrix parameters.

Symbol	Description	Value
$M_f$	Shape factor of the yield surface with mean effective stress	1.75
$\chi _f$	Shape factor of the yield surface	0.95
$M_g$	Critical state stress ratio in compression	1.75
$\chi _g$	Shape factor of the plastic potential	0.98
$\lambda ^*$	Slope of the isotropic compression line	0.32
$\kappa ^*$	Slope of the unloading-reloading line	0.06
$\nu _m$	Poisson ratio	0.20
$D_0$	Initial amplitude of distortional hardening	0.30
$D_1$	Decay rate of distortional hardening	2.00
$p_{\text {ref}}$	Reference pressure (kPa)	1.00

${*}$ In $\log {e} \text { to } \log {(p'/p_{\text {ref}})}$ space, where *e* is the void ratio.

## Data Availability

The datasets generated and analysed by the current study are available from the corresponding author upon reasonable request.
